# Harnessing enzyme plasticity for the synthesis of oxygenated sesquiterpenoids

**DOI:** 10.3762/bjoc.15.215

**Published:** 2019-09-17

**Authors:** Melodi Demiray, David J Miller, Rudolf K Allemann

**Affiliations:** 1 School of Chemistry, Cardiff University, Main Building, Park Place, Cardiff, CF10 3AT. United Kingdom

**Keywords:** artemisinin, amorphadiene synthase, oxygenated terpenoids, sesquiterpenoids, substrate engineering, terpenes

## Abstract

8-Methoxy-γ-humulene, (*E*)-8-methoxy-β-farnesene, 12-methoxy-β-sesquiphellandrene and 12-methoxyzingiberene can be synthesised in amorphadiene synthase-catalysed reactions from 8- and 12-methoxyfarnesyl diphosphates due to the highly plastic yet tightly controlled carbocationic chemistry of this sesquiterpene cyclase.

## Introduction

**Scheme 1:**
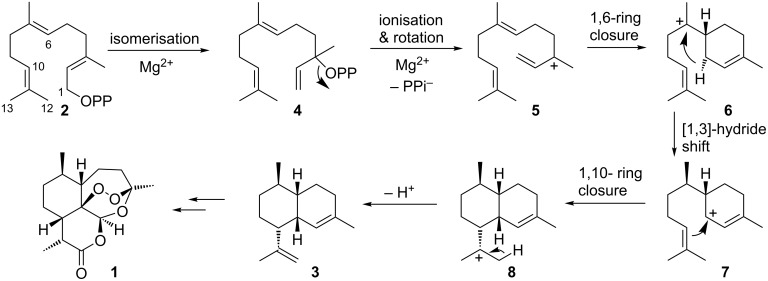
Mechanism of the ADS-catalysed conversion of FDP (**2**) to amorpha-4,11-diene (**3**), a biosynthetic precursor of artemisinin (**1**).

Amorphadiene synthase (ADS) from *Artemisia annua* is a key enzyme involved in the biosynthesis of the antimalarial sesquiterpene drug artemisinin (**1**) [1–4]. ADS catalyses the Mg^2+^-dependent conversion of farnesyl diphosphate (FDP, **2**) to amorpha-4,11-diene (**3**) with high regio- and stereochemical control (Scheme 1) [5–7]. The carbocationic reaction mechanism of ADS involves an isomerisation to nerolidyl diphosphate (NDP, **4**) followed by breakage of the carbon–oxygen bond to generate the allylic cation **5**. This allows rotation around the C2–C3 bond and 1,6-ring closure to form the bisabolyl cation (**6**). A [1,3]-hydride shift to form carbocation **7** and 1,10-ring closure yield the amorphyl cation (**8**). Finally, deprotonation generates amorpha-4,11-diene (**3**) [8–9].

Several sesquiterpene synthases including ADS accept FDP analogues containing a variety of heteroatoms and functional groups to generate unnatural sesquiterpenoids that are not easily accessible by conventional organic synthesis [10–19]. Creating novel sesquiterpenoids, not normally found in nature, is of great interest due to the important applications of terpenoids in healthcare and agriculture as well as the potential to tailor their properties to specific needs. For example, fluorinated derivatives of (*E*)-β-farnesene, a potent alarm pheromone for aphids [20–23], are more effective as pheromones than the parent compound, and finding high potency derivatives of (*E*)-β-farnesene may be of significant benefit in agriculture [24]. While (*S*)-germacrene D is a highly volatile but unstable olfactory signal that repels invertebrate arthropod pests (insects, ticks, mites) that affect humans and livestock as well as arable crops, (*S*)-14,15-dimethylgermacrene D acts as an attractant of aphids [10]. β-Sesquiphellandrene and α-curcumene are both found in turmeric (*Curcuma longa*) and have been shown to have anticancer activity [25–26]. The oxygenated α-curcumene and β-sesquiphellandrene derivatives α- and β-turmerone are reported to possess anticonvulsant properties and are used to treat epilepsy [27–28]. This array of important compounds shows the potential of generating novel sesquiterpenoids with desirable bio-properties.

ADS is a high fidelity sesquiterpene synthase that produces almost exclusively a single product. Its active site plasticity nevertheless allows the conversion of 12-hydroxy-FDP (**9**) to dihydroartemisinic aldehyde (**10**), a biosynthetic intermediate and valuable precursor in the synthesis of artemisinin [29].

## Results and Discussion

**Scheme 2:**
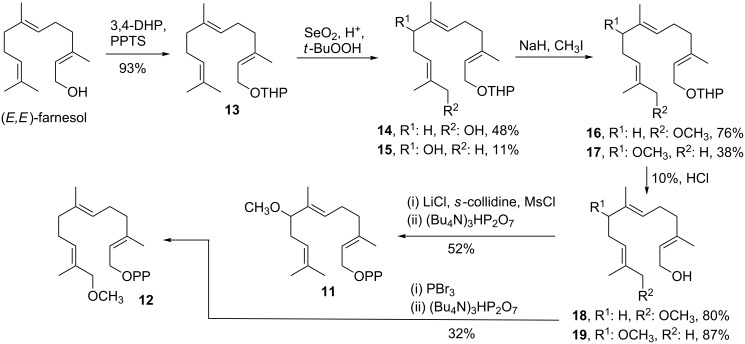
Synthesis of 8-methoxy-FDP (**11**) and 12-methoxy-FDP (**12**) (for full synthesis details see Supporting Information File 1).

Here we report that ADS accepts the bulkier FDP analogues 8-methoxy-FDP (**11**) and 12-methoxy FDP (**12**) as substrates, thereby opening up direct and efficient synthetic routes to oxidised sesquiterpenoids. This represents in essence a reversal of the biosynthetic pathways to oxidised sesquiterpenoids since cyclisation occurs after FDP ‘oxidation’. 8-Methoxy-FDP (**11**) and 12-methoxy-FDP (**12**) were both prepared in the same manner, beginning with a tetrahydropyranyl (THP) protection of (*E*,*E*)-farnesol to form **13** [29–30]. This was followed by a selenium dioxide oxidation that produced 12-hydroxyfarnesol (**14**) as the major product (48%, already published in [29]) in addition to 8-hydroxyfarnesol (**15**, 11%) [31]. Both of these alcohols were methylated with methyl iodide to yield **16** and **17** [32]. To produce the final FDP analogues, THP was removed to generate alcohols **18** and **19**, and subsequently diphosphorylated via halogenated intermediates (Scheme 2 and Supporting Information File 1) [33–35].

**Figure 1:**
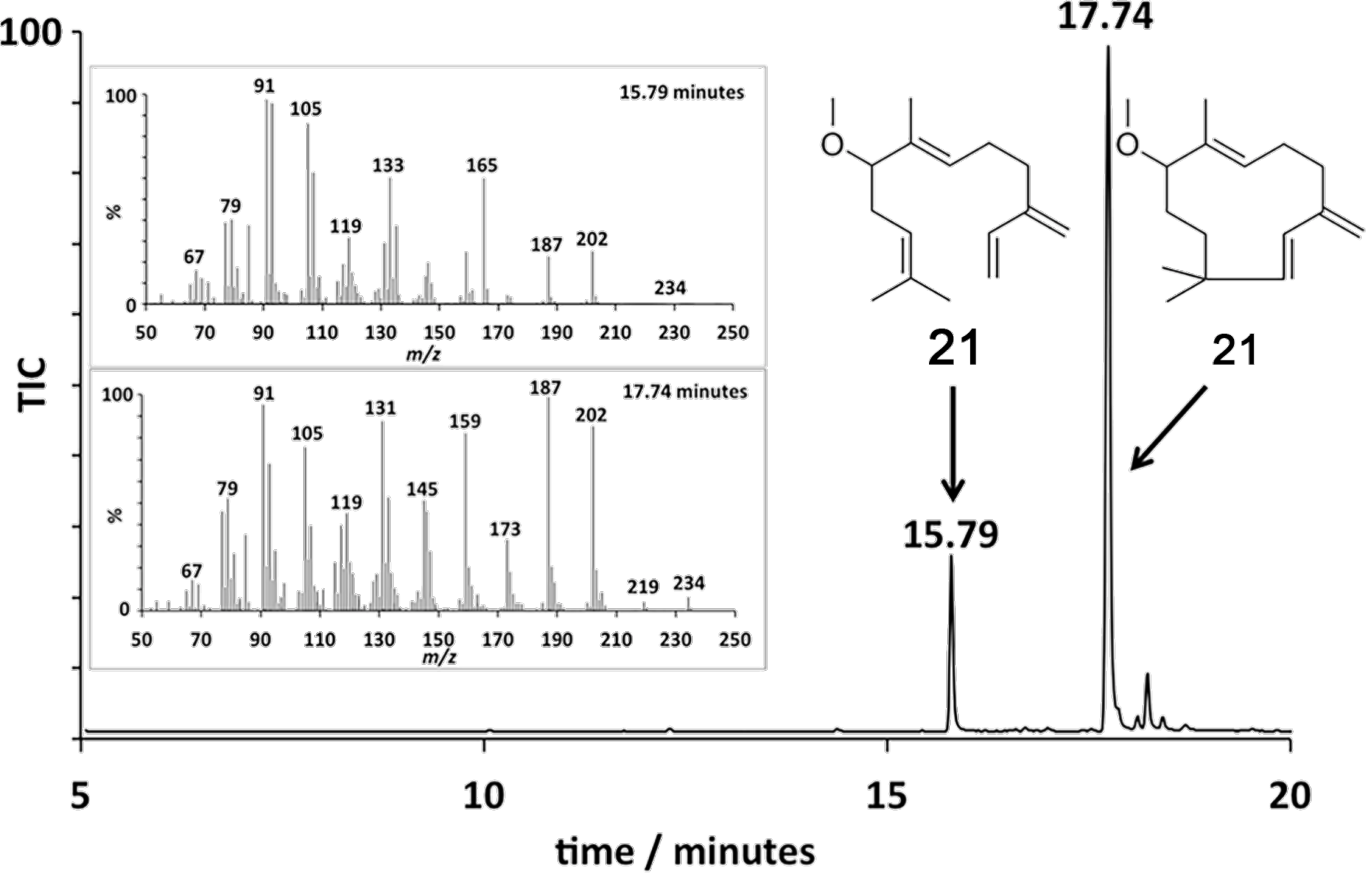
Total-ion chromatogram of the pentane extractable products formed in an incubation of ADS with 8-methoxy-FDP (**11**). Inset: EI^+^ Mass spectra of the eluted compounds **20** and **21**.

**Figure 2:**
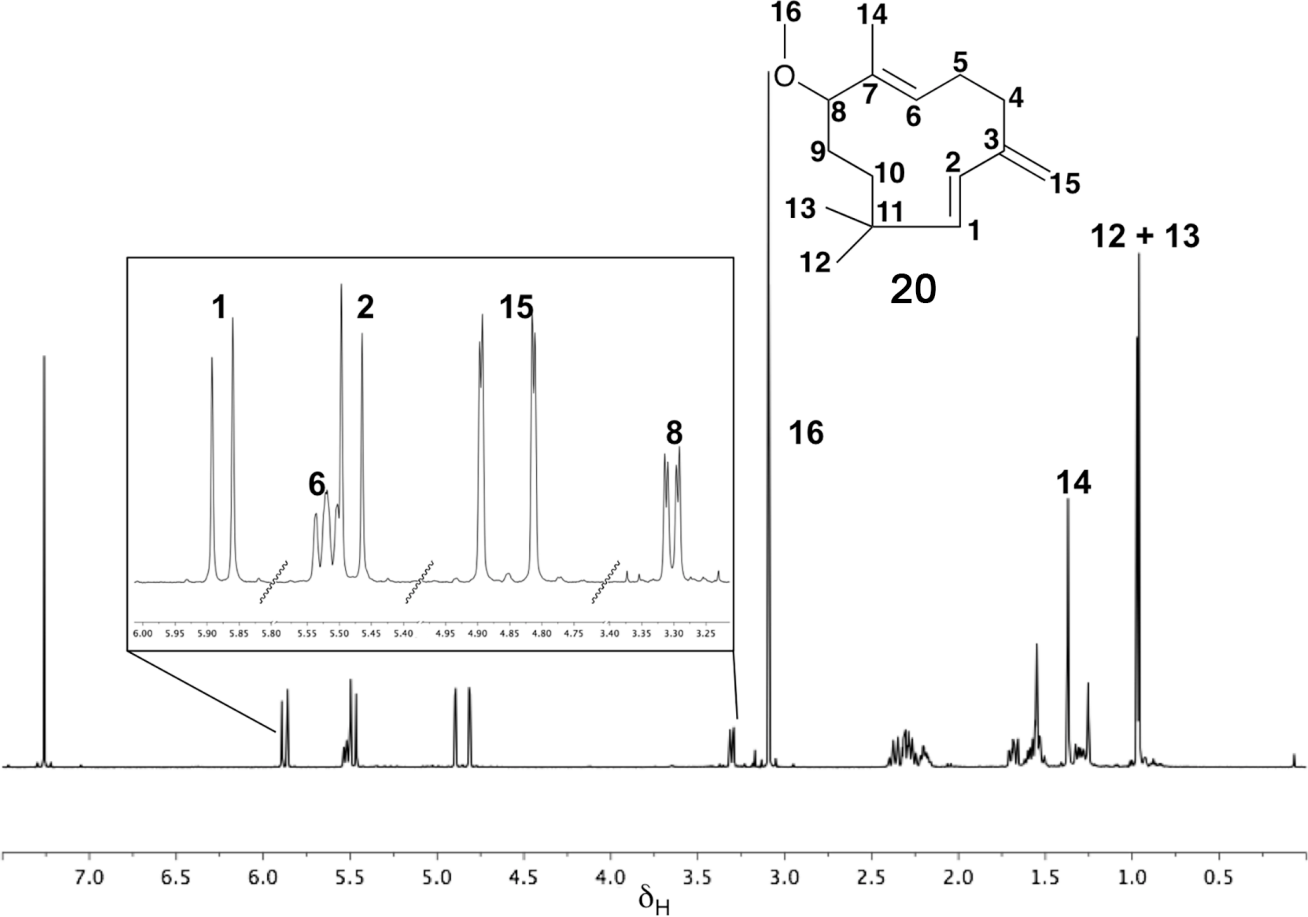
^1^H NMR spectrum (500 MHz, CDCl_3_) of the 8-methoxy-γ-humulene (**20**) generated by ADS from 8-methoxy-FDP (**11**).

GC–MS analysis (Figure 1) of the organic soluble products formed from 8-methoxy FDP (**11**) through ADS catalysis revealed the formation of a major sesquiterpenoid of molecular mass 234 (85%). No organic soluble products were detected when ADS was omitted from the incubation mixture. This product was identified as 8-methoxy-γ-humulene (**20**) by NMR spectroscopy and comparison of its ^1^H NMR spectrum (Figure 2) with that of 8-oxo-γ-humulene, a natural sesquiterpenoid isolated from the plant *Cineraria fruticulorum* [36]. The signals for H5 (δ_H_ = 5.44 ppm, d, *J*_H,H_ = 16.0 Hz), H6 (δ_H_ = 6.06, d, *J*_H,H_ = 16.0 Hz), H15 (δ_H_ = 5.00, bs) and H15’ (δ_H_ = 5.02, bs) of 8-oxo-γ-humulene [36] are in agreement with their corresponding equivalents in **20** (H2 (δ_H_ = 5.48, d, *J*_H,H_ = 16.0 Hz), H1 (δ_H_ = 5.88, d, *J*_H,H_ = 16.0 Hz), H15 (δ_H_ = 4.81, d, *J*_H,H_ = 2.5 Hz) and H15’ (δ_H_ = 4.89, d, *J*_H,H_ = 2.5 Hz)). The resonances for the methyl groups at C12 and C13 are also analogous, appearing as singlets at 1.03 ppm in 8-oxo-γ-humulene and 0.96 and 0.97 ppm in **20**. The identity of the minor product (15%) was confirmed as (*E*)-8-methoxy-β-farnesene (**21**) by GC co-elution and comparison of its mass spectrum with an authentic sample prepared by exposing (*E*)-β-farnesene synthase to diphosphate **11** (Supporting Information File 1). Further support for the structure of the minor product came from the excellent agreement of the diagnostic ^1^H NMR signals of **21** (H1 (δ_H_ = 5.25, d, *J*_H,H_ = 17.5 Hz), H1’ (δ_H_ = 5.06, d, *J*_H,H_ = 11.0 Hz), H2 (δ_H_ = 6.38, dd, *J*_H,H_ = 17.5 and 11.0 Hz), H15 (δ_H_ = 5.02, s), H15’ (δ_H_ = 5.00, s)) with those reported for the parent sesquiterpene (*E*)-β-farnesene (Supporting Information File 1) [37]. The proton on C6 (δ_H_ = 5.34, br, t) resonates further downfield for **21** compared to the corresponding proton of (*E*)-β-farnesene (δ_H_ = 5.17, t, *J*_H,H_ = 7.0 Hz) due to the presence of the methoxy group two carbon atoms away.

The ADS-catalysed 1,11-cyclisation of diphosphate **11** suggests that the 8-methoxy group prevents the formation of a conformation conducive to isomerisation to NDP (**4**, Scheme 1) and hence 1,6-cyclisation to generate a bisabolyl cation and the amorphane skeleton. Rather the active site conformations of **11** and cation **22** appear to enable a 1,11-cyclisation to **23**.

**Scheme 3:**
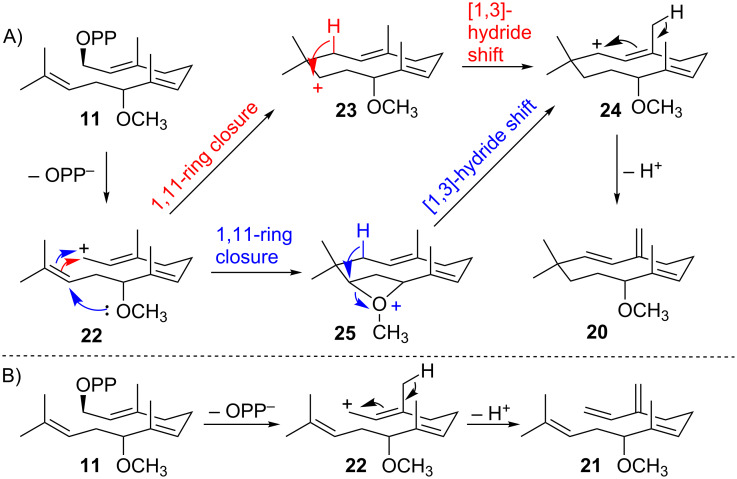
Potential mechanisms for the ADS-catalysed conversion of 8-methoxy-FDP (**11**) to 8-methoxy-γ-humulene (**20**) and (*E*)-8-methoxy-β-farnesene (**21**).

A subsequent [1,3]-hydride shift to cation **24** and deprotonation from C15 lead to 8-methoxy-γ-humulene (**20**, Scheme 3A). Alternatively, the nucleophilic 8-methoxy group could react at C10 to induce a fast 1,11-cyclisation, forming cation **25**, which effectively competes with the isomerization of **11** to 8-methoxy-NDP. A subsequent [1,3]-hydride shift leads to **24** (Scheme 3A). Direct deprotonation of **22** at C15 forms the minor reaction product (*E*)-8-methoxy-β-farnesene (**21**) (Scheme 3B). Due to the racemic nature of the starting diphosphate **11b** it is, however, also possible that each enzymatic product arises independently from the individual enantiomers.

**Figure 3:**
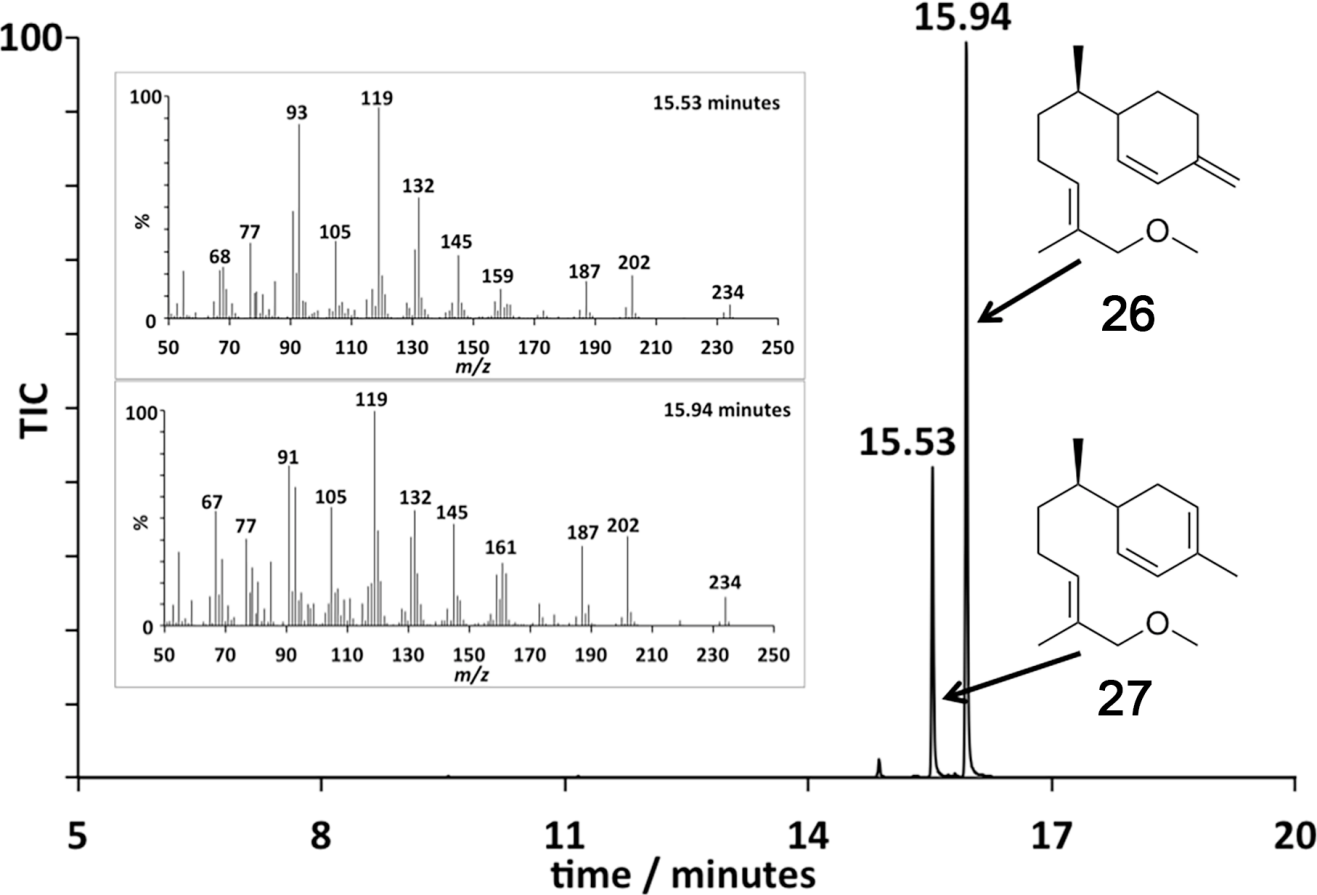
Total-ion chromatogram of the pentane extractable products formed in an incubation of ADS with 12-methoxy-FDP (**12**). Inset: Mass spectra of **26** and **27**.

**Figure 4:**
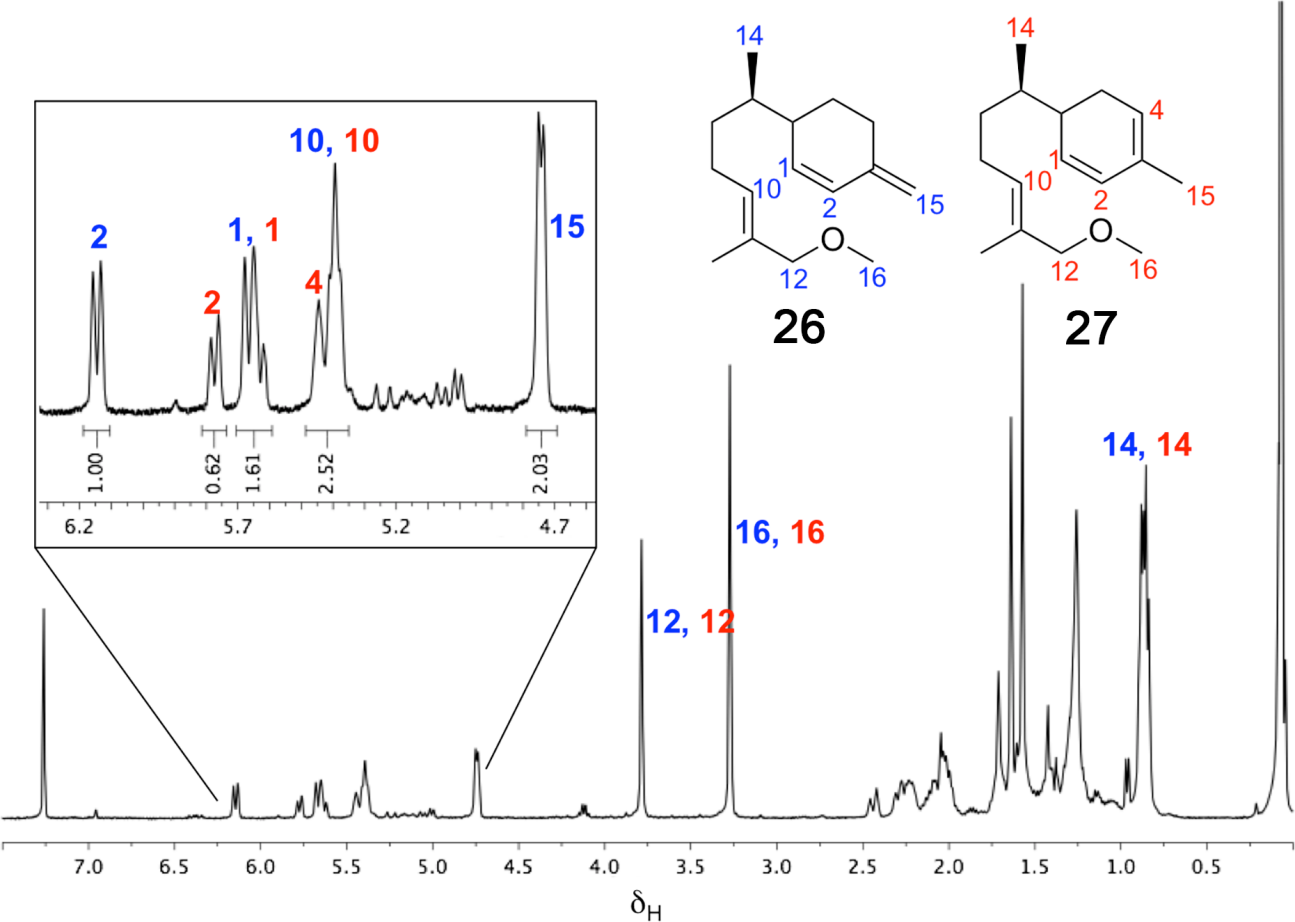
^1^H NMR spectrum (400 MHz, CDCl_3_) of 12-methoxy-β-sesquiphellandrene (**26**) and 12-methoxyzingiberene (**27**) produced by ADS from 12-methoxy-FDP (**12**).

GC–MS analysis of the organic soluble products generated from an incubation of ADS with 12-methoxy-FDP (**12**) revealed the formation of a 1:2.4 mixture of two sesquiterpenoids of mass *m/z* 234 (Figure 3). Again, no organic soluble products were detected when ADS was omitted from the incubation mixture. The ^1^H NMR spectrum of this product mixture (Figure 4) and comparison with the ^1^H NMR spectra of the bisabolyl-derived sesquiterpenes β-sesquiphellandrene [38–40] and zingiberene [40], a hydrocarbon with antifertility, antiviral and anticancer activity [41], suggested that the major compound was 12-methoxy-β-sesquiphellandrene (**26**), while the minor product was identified as 12-methoxyzingiberene (**27**). The two doublets observed at 0.85 and 0.87 ppm correspond to the C14H_3_ groups for both compounds. The ^13^C-DEPT-135 spectrum (see Supporting Information File 1) showed an inverted peak at ≈109 ppm, implying the presence of an olefinic CH_2_ that couples to the two overlapped doublets at 4.74 ppm. The integration of these doublets as 2 protons suggested that the exocyclic alkene was present in only one of the products. In the literature, this exocyclic alkene in β-sesquiphellandrene is observed at 4.72 ppm as a multiplet [39]. A triplet at 5.39 ppm that integrates for 2 protons can be assigned to H10 in both **26** and **27**, which resonates further downfield than in β-sesquiphellandrene and zingiberene due to the methoxy group positioned two carbons away. The olefinic protons H1 (δ_H_ = 5.67, d, *J*_H,H_ = 10.0 Hz) and H2 (δ_H_ = 6.14, d, *J*_H,H_ = 10.0 Hz) of **26** are in agreement with the equivalent protons H1 (δ_H_ = 5.66, dd, *J*_H,H_ = 10.0 and 2.5 Hz) and H2 (δ_H_ = 6.13, d, *J*_H,H_ = 10.0 Hz) in β-sesquiphellandrene. Similarly, the signals for the olefinic protons H1 (δ_H_ = 5.63), H2 (δ_H_ = 5.77) and H4 (δ_H_ = 5.45) in **27** correspond to the equivalent protons (δ_H_ = 5.61), H2 (δ_H_ = 5.57) and H4 (δ_H_ = 5.42) in zingiberene [40].

Mechanistically, isomerisation of **12** to the methoxy-NDP analogue **28** allows for an ADS-catalysed 1,6-cyclisation to the 12-methoxy-bisabolyl cation (**29**) followed by a [1,3]-hydride shift, which relocates the positive charge on C1 in **30**. With FDP and 12-hydroxy-FDP, a subsequent 1,10-ring closure has been proposed previously [29]. However, the presence of monocyclic products indicates that this second ring closure does not occur with 12-methoxy-FDP (**12**).

**Scheme 4:**
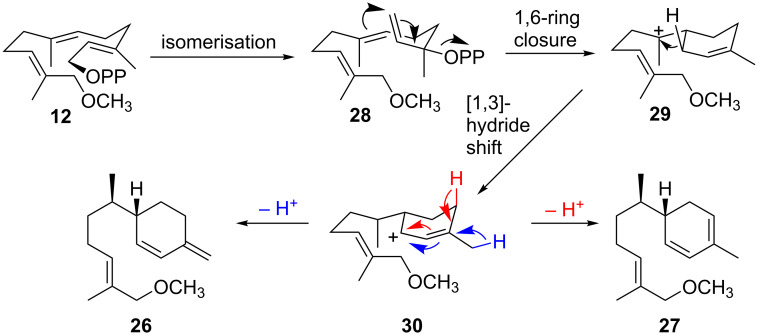
Possible mechanisms for the ADS-catalysed conversion of 12-methoxy-FDP (**12**) to 12-methoxy-β-sesquiphellandrene (**26**) and 12-methoxyzingiberene (**27**).

It is suggested that the 12-methoxy group enforces an orientation of the distal 10,11-double bond that is not conducive to the second ring closure. Subsequent deprotonation from C15 and C4 from intermediate **30** affords 12-methoxy-β-sesquiphellandrene (**26**) and 12-methoxyzingiberene (**27**), respectively (Scheme 4).

## Conclusion

In conclusion, the class I sesquiterpene cyclase amorphadiene synthase facilitates the efficient conversion of readily accessible synthetic methoxy-FDPs to sesquiterpenoids that may have applications in healthcare and agriculture. These results inform us of both the utility and limitations that non-natural functional groups have upon terpene cyclase-catalysed reaction cascades supporting the design of future biocatalytic syntheses. In particular, the presence of an ethereal oxygen atom containing π-acid functionality alongside its inductive withdrawal effect has a profound effect on the carbocationic reactivity of the intermediates. Of course a fully comprehensive interpretation of these results, regarding potentially interesting aspects such as anchimeric assistance is hampered by unknowns such as the conformation of binding to the enzyme and what effect the extra bulk of the substituents has upon the results observed, but nevertheless such empirical results will accumulate to inform future investigations. This reversal of the biosynthetic reaction order is expandable to other terpene synthases to generate libraries of unnatural sesquiterpenoids with a wide range of potential uses and applications across many areas of human activity.

## Supporting Information

File 1 Experimental part.
