# Supplementary material for: Harnessing enzyme plasticity for the synthesis of oxygenated sesquiterpenoids
Source: Beilstein J Org Chem. 2019 Sep 17;15:2184–90. doi: 10.3762/bjoc.15.215 (PMC6774066; doi:10.3762/bjoc.15.215)
Supplement: File 1 — Experimental part. [file Beilstein_J_Org_Chem-15-2184-s001.pdf]

BEILSTEIN JOURNAL OF ORGANIC CHEMISTRY

Supporting Information

for

Harnessing enzyme plasticity for the synthesis of oxygenated sesquiterpenoids

Melodi Demiray, David J. Miller and Rudolf K. Allemann

Beilstein J. Org. Chem. **2019,** *15,* 2184–2190. doi:10.3762/bjoc.15.215

Experimental part

License and Terms: This is a supporting information file under the terms of the Creative Commons Attribution License (http://creativecommons.org/licenses/by/4.0). Please note that the reuse, redistribution and reproduction in particular requires that the authors and source are credited.
The license is subject to the *Beilstein Journal of Organic Chemistry* terms and conditions: (https://www.beilstein-journals.org/bjoc)

Table of contents

1. General methods and materials S2

2. Synthetic procedures S3

3. Preparation of amorphadiene synthase (ADS) S11

4. Enzymatic incubations S14

5. GC–MS S18

6. NMR spectra S22

7. References S43

1. General methods and materials

For synthetic procedures, all chemicals and solvents were obtained from commercial vendors and used without further purification unless otherwise noted. Anhydrous tetrahydrofuran (THF), diethyl ether, toluene and acetonitrile were obtained from an MBraun SPS800 solvent purification system. Dichloromethane, and triethylamine were distilled from calcium hydride and potassium hydroxide under nitrogen respectively**.** TLC visualisations were performed with 4.2% ammonium molybdate and 0.2% ceric sulfate in 5% H_2SO_4 or UV light. Flash chromatography was performed according to the method of Still [1].

^{1}H and ^{13}C NMR spectra were measured on a Bruker Avance 500 NMR spectrometer or a Bruker Fourier 300 NMR spectrometer and are reported as chemical shifts in parts per million downfield from tetramethylsilane, multiplicity (s = singlet, d = doublet, t = triplet, q = quartet, m = multiplet), coupling (to the nearest 0.5 Hz) and assignment, respectively. GC–MS analyses of incubation products were performed on a Hewlett Packard 6890 GC apparatus fitted with a J&W scientific DB-5MS column (30 m × 0.25 mm internal diameter) and a Micromass GCT Premiere detecting in the range *m/z* 50–800 in the EI^+ mode with scanning once a second with a scan time of 0.9 s. Method: The program used an injection port temperature of 100 °C; split ratio 5:1; initial temperature 50 °C, hold 1 min, ramp of 4 °C/min to 150 °C hold 15 min, ramp of 20 °C/min to 250 °C hold 3 min. GC-FID analyses of incubation products were measured using an Agilent 7890A GC system with a manual injector and a SUPELCO Aztec CHIRALDEXTM B-DM silica capillary column. Carrier gas was helium (flow rate: 1 mL min^{-1}, split ratio 20:1) and the method used was isothermal, the oven temperature was held at 150 °C for 30 minutes. High-resolution ES^- mass spectra were measured on a Micromass LCT premiere XE spectrometer fitted with a Waters 1525 Micro binary

HPLC pump. The purity of final compounds were judged to be > 95% by TLC and/or GC analyses and NMR spectra analysis.

2. Synthetic procedures

Farnesyl diphosphate was synthesised as described by Davisson *et al.* [2]. Tetrahydropyranyl protected *(E*,*E*)-farnesol was prepared as previously described [3].

2.1. Preparation of 8-methoxyfarnesyl diphosphate (8-OMe FDP, 11).

(6*E*,10*E*)-2,6,10-Trimethyl-12-((tetrahydro-2*H*-pyran-2-yl)oxy)dodeca-2,6,10-trien-5-ol (15)

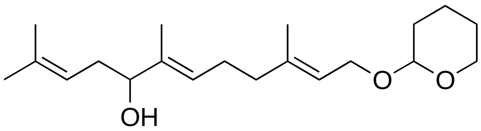

This compound was prepared in a similar manner to compound **14**, as described previously [3]. Selenium dioxide (0.11 g, 1.0 mmol), salicylic acid (0.14 g, 1.0 mmol), and *tert*-butyl hydroperoxide (1.6 mL, 16.3 mmol) were stirred in dichloromethane (20 mL) for 30 min at room temperature. The temperature was reduced to 0 °C before the addition of THP protected farnesol (1 g, 3.3 mmol). The reaction was left to stir for 24 h whilst remaining between 0–4 °C. Dichloromethane was removed using reduced pressure. *tert*-Butyl hydroperoxide was removed by co-evaporation with toluene (2 × 20 mL) under reduced pressure. The crude residue was dissolved in diethyl ether (20 mL) and washed with saturated aqueous sodium bicarbonate solution (50 mL). The layers were separated and the aqueous layer was extracted with diethyl ether (5 × 20 mL). The combined organic layers were washed with brine (20 mL) and dried over anhydrous magnesium sulfate before being filtered under gravity. The solvent was

removed under reduced pressure and the crude oil was purified by flash chromatography on silica gel (9:1, hexane/ethyl acetate) to give the title compound as a colourless oil (116 mg, 11%) plus compound **14** as the major product (48%, charaterisation as previously described [3]).

^{1}H NMR (300 MHz, $CDCl_3$): δ 5.39-5.34 (2 H, m, 2 x $C{=}C\underline{H}CH_2$), 5.08 (1 H, t, $J_{H,H}$ = 7.0, $C{=}C\underline{H}CH_2$), 4.63 (1 H, m, $OC\underline{H}O$), 4.25 (1 H, dd, $J_{H,H}$ = 12.0 and 6.5, $CHC\underline{H_A}O$), 4.05-3.95 (2 H, m, $CHC\underline{H_B}O$ and $C\underline{H}OH$), 3.98 (1 H, t, $J_{H,H}$ = 7.5, $C\underline{H}OH$), 3.92-3.48 (2 H, m, $OC\underline{H_2}CH_2$), 2.34-2.04 (6 H, m, $CHC\underline{H_2}CH$ and $CHC\underline{H_2}C\underline{H_2}$), 1.88-1.50 (6 H, m, $CHC\underline{H_2}C\underline{H_2}C\underline{H_2}$), 1.72, 1.68, 1.63 and 1.61 (4 x 3 H, 4 x s, 4 x $C\underline{H_3}$). **^{13}C NMR** (125 MHz, $CDCl_3$): δ 140.0, 137.3 and 134.8 (3 x $CH_3\underline{C}CH$), 125.9, 121.1 and 120.4 (3 x $CH_3C\underline{C}H$), 98.1 ($O\underline{C}HO$), 63.9 ($\underline{C}HOH$), 63.9 and 62.5 (2 x $\underline{C}H_2O$), 39.4 ($CH_3C\underline{C}H_2$), 34.3 ($\underline{C}H_2CHOH$), 30.9 ($CH_2OCH\underline{C}H_2$), 26.1 ($OCH_2\underline{C}H_2CH_2$), 25.7 ($OCH_2CH_2\underline{C}H_2$), 19.8, 18.2, 16.6 and 11.9 (4 x $\underline{C}H_3$). **HRMS** (ES^+): calculated for $(C_{20}H_{34}O_3Na)^+$: 345.2406, found: 345.2418.

2-(((2*E*,6*E*)-8-Methoxy-3,7,11-trimethyldodeca-2,6,10-trien-1-yl)oxy)tetrahydro-2*H*-pyran (17)

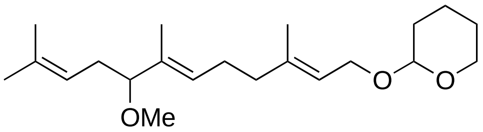

To a stirred suspension of sodium hydride (0.40 g, 60% in mineral oil, 9.3 mmol) in anhydrous THF (30 mL), was added a solution of the above 8-hydroxyfarnesyl-THP (0.50 g, 1.6 mmol) in anhydrous THF (20 mL) followed by the addition of methyl iodide (1.5 mL, 23 mmol) 15 min later. The reaction was left to stir under argon for 24 h whereupon TLC analysis (8:2, hexane/ethyl acetate) showed full consumption of starting material. The reaction was quenched with 10% hydrochloric acid (50 mL) and

the layers were separated. The aqueous layer was washed with diethyl ether (4 × 20 mL) and the pooled extracts were washed with 5% sodium hydroxide (3 × 10 mL) and dried over anhydrous magnesium sulfate. The solvent was removed under reduced pressure. The crude oil was purified by flash chromatography on silica gel (4:1, hexane: ethyl acetate) to give the title compound as a colourless oil (0.2 g, 38%).

^{1}H NMR (300 MHz, $CDCl_3$): δ 5.37 (1 H, t, $J_{H,H}$ = 6.5, $C{=}C\underline{H}CH_2$), 5.30 (1 H, t, $J_{H,H}$ = 6.5, $C{=}C\underline{H}CH_2$), 5.03 (1 H, t, $J_{H,H}$ = 7.0, $C{=}C\underline{H}CH_2$), 4.62 (2 H, t, $J_{H,H}$ = 4.0, $OC\underline{H}O$), 4.27-3.98 (2 H, m, $CCHC\underline{H_2}O$), 3.92-3.48 (2 H, m, $OC\underline{H_2}CH_2$), 3.41 (1 H, t, $J_{H,H}$ = 7.0, $C\underline{H}OCH_3$), 3.16 (3 H, s, $OC\underline{H_3}$), 2.34-2.04 (6 H, m, $CCHC\underline{H_2}CH$ and $CCHC\underline{H_2}C\underline{H_2}$), 1.75-1.52 (6 H, m, $OC\underline{H_2}C\underline{H_2}C\underline{H_2}$), 1.68, 1.59, and 1.52 (2 x 3 H and 1 x 6 H, 3 x s, 4 x $CC\underline{H_3}$). **^{13}C NMR** (125 MHz, $CDCl_3$): δ 134.5 and 132.9 (3 x $\underline{C}{=}CH$), 128.5, 121.2 and 120.9 (3 x $C{=}\underline{C}H$), 98.1 ($O\underline{C}HO$), 87.6 ($\underline{C}HOCH_3$), 63.9 and 62.5 ($O\underline{C}H_2$), 55.9 ($O\underline{C}H_3$), 39.5 ($CH_3C\underline{C}H_2$), 32.7 ($CCH\underline{C}H_2CH$), 30.9 ($CH_2OCH\underline{C}H_2CH_2$), 26.1 ($CH_3CCH_2\underline{C}H_2$), 25.9 ($CHC\underline{C}H_3$), 25.7 ($OCH_2\underline{C}H_2CH_2$), 19.8 ($OCH_2CH_2\underline{C}H_2$), 18.1, 16.6 and 10.7 (3 x $C\underline{C}H_3$). **HRMS** (ES^+): calculated for $(C_{21}H_{36}O_3 + [Na])^+$: 359.2562, found: 359.2554

(2*E*,6*E*)-8-Methoxy-3,7,11-trimethyldodeca-2,6,10-trien-1-ol (19)

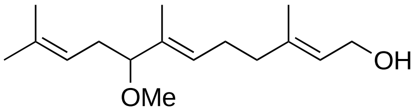

To a solution of the above 8-methoxy farnesyl THP (0.20 g, 1.6 mmol) in anhydrous THF (10 mL) was added a solution of 10% hydrochloric acid (7 mL) and was left to stir for 4 h. The reaction was quenched with saturated sodium bicarbonate solution (10 mL) and the biphasic layers were separated. The aqueous layer was washed with diethyl ether (4 × 10 mL) and the pooled extracts were washed with brine (10 mL) before being dried over anhydrous magnesium sulfate, filtered under gravity and

concentrated under reduced pressure. The crude oil was purified by flash chromatography on silica gel (4:1, hexane/ethyl acetate) to give the title compound as colourless oil (130 mg, 87%).

^{1}H NMR (300 MHz, $CDCl_3$): δ 5.42 (1 H, t, $J_{H,H}$ = 7.0, $C{=}C\underline{H}$), 5.30 (1 H, t, $J_{H,H}$ = 7.0, $C{=}C\underline{H}$), 5.02 (1 H, t, $J_{H,H}$ = 7.0, $C{=}C\underline{H}$), 4.15 (2 H, d, $J_{H,H}$ = 7.0, $C\underline{H_2}OH$), 3.41 (1 H, t, $J_{H,H}$ = 7.0, $C\underline{H}OCH_3$), 3.16 (3 H, s, $OC\underline{H_3}$), 2.34-2.04 (6 H, m, $CHC\underline{H_2}CHO$ and $CHC\underline{H_2}C\underline{H_2}$), 1.68 (6 H, s, 2 x $CC\underline{H_3}$), 1.59 and 1.52 (2 x 3 H, 2 x s, 2 x $CC\underline{H_3}$). **^{13}C NMR** (125 MHz, $CDCl_3$): δ 139.6, 134.5 and 133.0 (3 x $\underline{C}{=}CH$), 128.4, 123.8 and 120.8 (3 x $C{=}\underline{C}H$), 87.6 ($\underline{C}HOCH_3$), 59.6 ($\underline{C}H_2OH$), 55.9 ($O\underline{C}H_3$), 39.4 ($CH_3C\underline{C}H_2$), 32.6 ($\underline{C}H_2CHO$), 26.0 ($CH_3CCH_2\underline{C}H_2$), 26.0, 18.1, 16.4 and 10.7 (4 x $C\underline{C}H_3$). **HRMS** (ES^+): calculated for $(C_{16}H_{28}O_2 + [Na])^+$: 275.1987, found: 275.1978.

8-Methoxyfarnesyl diphosphate (11)

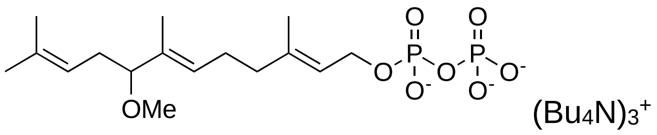

To a cold, stirred suspension (0 °C) of the above 8-methoxyfarnesol (0.13 g, 0.59 mmol), lithium chloride (87 mg, 2.0 mmol) and *s*-collidine (0.40 mL, 3.0 mmol) in anhydrous DMF (10 mL) was added methanesulfonyl chloride (0.026 mL, 0.34 mmol), under nitrogen. The solution was stirred for 3 h. The resulting solution was poured into cold water (30 mL) and extracted with diethyl ether (4 × 10 mL). Combined organic extracts were washed with saturated aqueous copper (II) sulfate solution (3 × 20 mL), water (2 × 20 mL) and saturated aqueous ammonium bicarbonate solution (2 × 20 mL). The resulting solution was dried over anhydrous magnesium sulfate, filtered under gravity and concentrated under reduced pressure to give the crude chloride intermediate. To a stirred solution of $(Bu_4N)_3HP_2O_7$ (0.9 g, 1.0 mmol) in anhydrous

acetonitrile (5 mL) under N_2 was added the solution of the crude chloride in acetonitrile (3 mL). This was left to stir for 48 h at room temperature. The solvent was removed under reduced pressure and the resulting crude oil was purified by flash chromatography on silica gel (6:2.5:0.5, isopropanol/water/ammonium hydroxide). Fractions that contained the diphosphate as judged by comparison with an authentic FDP standard by TLC (6:3:1, isopropanol: water: ammonium hydroxide) were collected. The isopropanol was removed under reduced pressure and the remaining solution was lyophilized to give the title compound as colourless oil (306 mg, 52%).

^{1}H NMR (300 MHz, $CDCl_3$): δ 5.40 (2 H, m, 2 x $C{=}C\underline{H}$), 5.05 (1 H, t, $J_{H,H}$ = 7.5, $C{=}C\underline{H}$), 4.46 (2 H, m, $C\underline{H_2}O$), 3.61 (1 H, t, $J_{H,H}$ = 7.0, $C\underline{H}OCH_3$), 3.19-3.14 (27 H, m, 12 x $NC\underline{H_2}CH_2CH_2CH_3$ and $CHOC\underline{H_3}$), 2.35-2.06 (6 H, m, $CHC\underline{H_2}CH$ and $CHC\underline{H_2}C\underline{H_2}$), 1.69-1.51 (36 H, m, 12 x $NCH_2C\underline{H_2}CH_2CH_3$ and 4 x CH_3), 1.36-1.29 (24 H, m, 12 x $NCH_2CH_2C\underline{H_2}CH_3$), 0.92 (36 H, t, $J_{H,H}$ = 7.5, 12 x $NCH_2CH_2CH_2C\underline{H_3}$). **^{31}P NMR** (121 MHz, D_2O): δ -7.62 (m), -7.88 (d, J = 17.0). **HRMS** (ES^-): calculated for $(C_{16}H_{30}O_8P_2 - [H])^-$: 411.1338, found: 411.1342.

2.2 Preparation of 12-methoxyfarnesyl diphosphate (12-OMe FDP, 12).

2-(((2*E*,6*E*,10*E*)-12-Methoxy-3,7,11-trimethyldodeca-2,6,10-trien-1-yl)oxy)tetrahydro-2*H*-pyran (16)

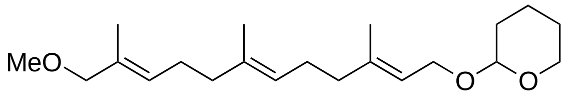

To a stirred suspension of sodium hydride (0.14 g, 60% in mineral oil, 3.7 mmol) in anhydrous THF (5 mL), was added a solution of **14** (prepared as published previously [3]) (0.2 g, 0.6 mmol) in anhydrous THF (5 mL) followed by the addition of methyl

iodide (1.5 mL, 23 mmol) after 15 min. The reaction was left to stir under argon for 24 h whereupon TLC analysis (8:2, hexane/ethyl acetate) showed full consumption of starting material. The reaction was quenched with 10% hydrochloric acid (10 mL) and the layers were separated. The aqueous layer was washed with diethyl ether (4 × 10 mL) and the pooled extracts were washed with 5% sodium hydroxide (3 × 10 mL) and dried over anhydrous magnesium sulfate. The solvent was removed under reduced pressure and the crude oil was purified by flash chromatography on silica gel (4:1, hexane/ethyl acetate) to give the title compound as a colourless oil (158 mg, 76%).

^{1}H NMR (300 MHz, $CDCl_3$): δ 5.40-5.33 (2 H, m, 2 x C=C$\underline{H}$), 5.11 (1 H, t, $J_{H,H}$ = 7.0, C=C$\underline{H}$), 4.62 (2 H, t, $J_{H,H}$ = 4.0, OC$\underline{H}$O), 4.24 (1 H, dd, $J_{H,H}$ = 12.0 and 6.5, CHC$\underline{H_A}$O), 4.02 (1 H, dd, $J_{H,H}$ = 12.0 and 7.5, CHC$\underline{H_B}$O), 3.93-3.47 (2 H, m, OC$\underline{H_2}$$CH_2$), 3.77 (2 H, s, C$\underline{H_2}OCH_3$), 3.26 (3 H, s, OC$\underline{H_3}$), 2.15-1.99 (8 H, m, 2 x CHC$\underline{H_2}$C$\underline{H_2}$), 1.71-1.54 (6 H, m, OC$\underline{H_2}$C$\underline{H_2}$C$\underline{H_2}$), 1.68, 1.63, and 1.60 (3 x 3 H, 3 x s, 3 x CC$\underline{H_3}$). **^{13}C NMR** (125 MHz, $CDCl_3$): δ 140.4, 135.1 and 132.1 (3 x $\underline{C}$=CH), 128.4, 124.3 and 120.8 (3 x C=$\underline{C}$H), 98.0 (O$\underline{C}$HO), 78.90 ($\underline{C}H_2OCH_3$), 63.84 and 62.49 (2 x CHO$\underline{C}H_2$), 51.69 (O$\underline{C}H_3$), 39.79 and 39.5 (2 x CH_3C$\underline{C}H_2$), 30.9 (OCH$\underline{C}H_2$), 26.5 and 26.4 (2 x CCH$\underline{C}H_2CH_2$), 25.7 ($OCH_2\underline{C}H_2CH_2$), 19.82 ($OCH_2CH_2\underline{C}H_2$), 16.6, 16.2 and 14.0 (3 x C$\underline{C}H_3$). **HRMS** (AP$^+$): calculated for $(C_{21}H_{36}O_3Na)^+$: 359.2549, found: 359.2562.

(2*E*,6*E*,10*E*)-12-Methoxy-3,7,11-trimethyldodeca-2,6,10-trien-1-ol (18)

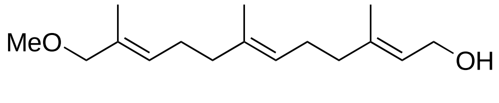

To a stirred solution of the above 12-methoxyfarnesyl THP (0.15 g, 0.45 mmol) in methanol (5 mL) was added *p*-toluenesulfonic acid (9.0 mg, 45 μmol) and stirred at room temperature for 1 h until TLC analysis (8:2, hexane: ethyl acetate) confirmed complete consumption of starting material. The solvent was removed under reduced pressure and the residual oil was dissolved with diethyl ether (10 mL) and washed with

saturated aqueous sodium bicarbonate solution (3 × 10 mL) and brine (10 mL). The resulting solution was dried over anhydrous magnesium sulfate, filtered under gravity and concentrated under reduced pressure to give the title compound as a colourless oil (91 mg, 80%). The product was judged by ^{1}H NMR spectroscopy to be sufficiently pure to carry forward without further purification.

^{1}H NMR (400 MHz, $CDCl_3$): δ 5.43-5.36 (2 H, m, 2 x $C{=}C\underline{H}$), 5.11 (1 H, t, $J_{H,H}$ = 7.0, $C{=}C\underline{H}$), 4.15 (2 H, d, $J_{H,H}$ = 7.0, $C\underline{H_2}OH$), 3.78 (2 H, s, $C\underline{H_2}OCH_3$), 3.27 (3 H, s, $CH_2OC\underline{H_3}$), 2.14-2.00 (8 H, m, 2 x $CHC\underline{H_2}C\underline{H_2}$), 1.68, 1.63, and 1.60 (3 x 3 H, 3 x s, 3 x $CC\underline{H_3}$). **^{13}C NMR** (125 MHz, $CDCl_3$): δ 139.8, 135.2 and 132.1 (3 x $\underline{C}{=}CH$), 128.2, 124.2 and 123.6 (3 x $C{=}\underline{C}H$), 78.9 ($\underline{C}H_2OCH_3$), 59.6 ($\underline{C}H_2OH$), 57.5 ($O\underline{C}H_3$), 39.7 and 39.4 (2 x $CH_3C\underline{C}H_2$), 26.4 and 26.4 ($CCH\underline{C}H_2CH_2$), 16.5, 16.2 and 14.0 (3 x $C\underline{C}H_3$). **HRMS** (ES^+): calculated for $(C_{16}H_{28}O_2Na)^+$: 275.1987, found: 275.1982.

(2*E*,6*E*,10*E*)-12-Bromo-1-methoxy-2,6,10-trimethyldodeca-2,6,10-triene

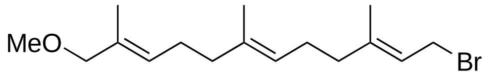

To a stirred solution of the above alcohol (0.66 g, 2.6 mmol) in anhydrous THF (10 mL) at −10 °C, was added phosphorous tribromide (0.12 mL, 1.3 mmol), and this was left to stir for 1 h. When TLC analysis (8:2, hexane/ethyl acetate) confirmed complete consumption of starting material, the phosphorous tribromide and THF were removed under reduced pressure. The residual product was diluted with diethyl ether (10 mL) and washed with saturated aqueous ammonium bicarbonate solution (3 × 20 mL) and brine (10 mL). The resulting solution was dried over anhydrous magnesium sulfate, filtered under gravity and concentrated under reduced pressure to give the title compound as a colourless oil (820 mg, 100%). The product was judged by ^{1}H NMR spectroscopy to be sufficiently pure to carry forward without further purification.

^{1}H NMR (300 MHz, $CDCl_3$): δ 5.53 (1 H, t, $J_{H,H}$ = 8.5, C<u>H</u>CH_2Br), 5.38 (1 H, t, $J_{H,H}$ = 7.0, C=C<u>H</u>), 5.09 (1 H, m, C=C<u>H</u>), 4.02 (2 H, d, $J_{H,H}$ = 8.5, C<u>H_2</u>Br), 3.78 (2 H, s, C<u>H_2</u>OCH_3), 3.27 (3 H, s, CH_2OC<u>H_3</u>), 2.18-2.00 (8 H, m, 2 x CHC<u>H_2</u>C<u>H_2</u>), 1.73, 1.64, and 1.60 (3 x 3 H, 3 x s, 3 x CC<u>H_3</u>).

12-Methoxyfarnesyl diphosphate (12)

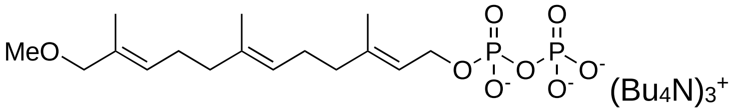

To a stirred solution of the above 12-methoxyfarnesol (0.82 g, 2.6 mmol) in anhydrous acetonitrile (10 mL) was added $(Bu_4N)_3HP_2O_7$ (4.7 g, 5.2 mmol). The reaction was left for 48 h to stir under nitrogen. The solvent was removed under reduced pressure and the resulting crude oil was purified by flash chromatography on silica gel (6:2.5:0.5, isopropanol/water/ammonium hydroxide). Fractions that contained the diphosphate as judged by comparison with an authentic FDP standard by TLC (6:3:1, isopropanol/water/ammonium hydroxide) were collected. The isopropanol was removed under reduced pressure and the remaining solution was lyophilized to give the title compound as colourless oil (900 mg, 32%).

^{1}H NMR (500 MHz, $CDCl_3$): δ 5.36-5.32 (2 H, m, 2 x C=C<u>H</u>), 5.07 (1 H, t, $J_{H,H}$ = 6.5, C=C<u>H</u>), 4.45 (2 H, t, $J_{H,H}$ = 5.5, CHC<u>H_2</u>O), 3.74 (2 H, s, C<u>H_2</u>OCH_3), 3.31-3.28 (24 H, m, 12 x NC<u>H_2</u>$CH_2CH_2CH_3$), 3.21 (3 H, s, CH_2OC<u>H_3</u>), 2.10-1.90 (8 H, m, 2 x CHC<u>H_2</u>C<u>H_2</u>), 1.61-1.54 (33 H, m, 12 x NCH_2C<u>H_2</u>CH_2CH_3 and 3 x CC<u>H_3</u>), 1.45-1.38 (24 H, m, 12 x NCH_2CH_2C<u>H_2</u>CH_3), 0.94 (36 H, t, $J_{H,H}$ = 7.5, 12 x $NCH_2CH_2CH_2$C<u>H_3</u>). **^{31}P NMR** (121 MHz, $CDCl_3$): δ -7.40 (d, $J_{P,P}$ = 17.0), -7.73 (d, $J_{P,P}$ = 17.0). **HRMS** (ES^-): calculated for $(C_{16}H_{29}O_8P_2)^-$: 411.1338, found: 411.1336.

3. Preparation of amorphadiene synthase (ADS)

ADS was prepared as described previously [3].

Subcloning the ADS cDNA into pET21d plasmid. A cDNA encoding amorphadiene synthase (ADS) from *Artemisia annua* was obtained from the gene bank (JF951730). The cDNA was supplied in a pTrc99a vector between the NcoI and BamHI restriction sites (pTrc-ADS). pET21d vector and pTrc-ADS were digested with *Nco*I and *BamH*I restriction endonucleases (0.1 μL of each enzyme, 1 μL of buffer, 10 μL of plasmid, 1 h, 37 °C), and the fragments corresponding to an open pET21d and ADS gene were ligated using T4 DNA ligase following the manufacturer's protocol (1:2 molar ratio of pET: ADS, 0.1 μL enzyme, 2 μL of buffer, H_2O to make total volume to 20 μL) to give a new plasmid pET21d-ADS. Supercompetent *E. coli* XL1-blue cells were transformed with 5 μL of ligated DNA and stored on ice (30 minutes) before being heat shocked in a water bath (40 °C, 40 s) and placed back into the ice for another 2 min. LB medium (1 mL) was added and the solution was shaken for 60 min (37 °C, 150 rpm). The cells were harvested by centrifugation (3400*g*, 1 minute) and spread on an agar plate containing ampicillin (100 μg/mL) after resuspending in a minimum amount of buffer. Plates were incubated overnight at 37 °C and then stored at 4 °C. A single colony from the agar plate was used to inoculate 15 mL of LB medium containing ampicillin (100 μg/mL). The culture was incubated overnight (37 °C) and the following day centrifuged (3220*g*, 8 min). The pellet was purified using a QIAprep Spin Miniprep Kit (QIAprep Miniprep Handbook-2005). The resulting 50 μL DNA solution was stored at −20 °C. Ligation of the ADS gene into the pET21d vector was confirmed by DNA sequence analysis from Eurofin.

Introduction of C-terminal 6xHis tag into ADS. A single nucleotide deletion was required to bring the 6xHis coding sequence of pET21d in frame with the ADS coding sequence. A Quickchange site-directed mutagenesis kit was used to introduce the desired deletion according to the manufacturer's instructions. The primers used for the deletion were as follows: 5'-CGATGTCCATCTGTCCCGGGGATCC-3' and 5'-GCTACAGGTAGACAGGGCCCCTAGG-3'. Polymerase chain reaction (PCR) (4 h) was carried out. Samples were then digested with *Dpn*I (1 h, 37 °C).

Plasmids were transformed into *E. coli* XL1-blue cells. A colony from the plate was selected and used to inoculate 15 mL of LB medium containing ampicillin (100 μg/mL). The resulting overnight cultures were centrifuged (3220*g*, 8 min) and the resulting pellets were purified using *QIAprep Spin Miniprep kit* (QIAprep Miniprep Handbook-2005). The resulting 50μL DNA solutions were stored at −20 °C. DNA was sequenced to confirm that the site-directed mutagenesis had been successful.

Transformation of competent cells. Plasmid pET21dADS was transformed into competent *E. coli* BL21-(DE3)-Codon Plus RP (EcBL21RP). The plasmid solution (1 μl) was added to the cell suspension (50 μL) and was stored on ice (30 min) before being heat shocked in a water bath (42 °C, 40 s) and placed back into the ice for another 2 min. LB medium (1 mL) was added and the solution was shaken for 60 min (37 °C, 150 rpm). The cells were then harvested by centrifugation (3300*g*, 1 minute) and spread on an agar plate containing ampicillin (100 μg/mL) after resuspending in a minimum amount of buffer. Plates were incubated overnight at 37 °C and then stored at 4 °C.

Expression of ADS gene. A single colony from the agar plate of *Ec*BL21RP cells was used to inoculate LB medium (100 mL) containing ampicillin (100 μg/ml). The culture was shaken overnight (37 °C, 150 rpm). The resulting cell suspension was used to inoculate LB medium (6 × 500 mL, 10 mL of cell suspension in each flask) containing ampicillin (100 μg/mL). The cells were grown (37 °C, 150 rpm) until the OD_{600} reached 0.5. Isopropyl β-D-1-thiogalactopyranoside (IPTG) (0.048 g, 0.4 mM) was added to each culture and shaking continued (20 °C, 150 rpm) for an additional 6 h. The cells were harvested by centrifugation (4500*g*, 10 minutes) and the resulting pellets were stored at −20 °C.

Purification of ADS. Cell pellets were re-suspended in 50 mL of cell lysis buffer (50 mM Tris-Base, 500 mM NaCl, 5 mM imidazole, 20 mM βME, 10% (v/v) glycerol, pH 8). Lysozyme (0.5 mg/mL) was added and the mixture was left to stir for 1 h at 5 °C. Cells were disrupted by sonication at 5 °C (38% amplitude for 3 min with 5 s on/10 s off cycles) and the resulting suspension was centrifuged at 5 °C (17000*g*, 30 min) Once SDS-polyacrylamide gel electrophoresis confirmed that the majority of the protein was found in the solution, the pellet was discarded and the supernatant solution was loaded onto a Ni Sepharose™ 6 Fast Flow column (12 mL, the column was eluted under gravity controlled drip flow). After 15 minutes, the flow through was eluted and the column was washed with 10 CV of cell lysis buffer followed by a gradient from 5 to 300 mM imidazole (40 CV). Fractions were analyzed by SDS-PAGE. The protein eluted at 100 mM imidazole. The fractions corresponding to a molecular weight of 56000 were pooled, dialyzed overnight against 25 mM HEPES, 100 mM NaCl, 1 mM DTT, pH 7.5 (MWCO 30000 Da) and then concentrated to a final volume of ≈10

mL (AMICON system, YM 30). Solutions were stored at 4 °C. The concentration of protein was estimated using the method of Bradford [4,5].

4. Enzymatic incubations

General procedure for analytical incubations of FDP (2), 8 OMe FDP (11) and 12 OMe FDP (12) with purified ADS. As in the procedure described previously [3] ADS (1 μM) and substrate (0.4 mM) were both incubated in 250 μL of incubation buffer (20 mM HEPES, 5 mM β-ME, 5 mM $MgCl_2$, pH 7.5), which was overlaid with 1 mL of pentane and left to gently shake overnight at room temperature. The organic layer was separated and analyzed by GC–MS.

General procedure for preparative scale incubations of FDP (2), 8 OMe FDP (11) and 12 OMe FDP (12) with purified ADS. To minimize losses of volatile sesquiterpenoid products during incubation and workup, reactions were performed in Schott bottles. Incubations contained incubation buffer (200 mL), substrate (0.4 mM) and enzyme (1 μM). The incubation was then overlaid with 10 mL of pentane. The incubation solution was gently agitated for 24 h at room temperature. The following day, the pentane layer was then separated and the aqueous layer was washed with an additional extraction of pentane (2 × 10 mL). The pooled pentane extracts were concentrated under reduced pressure and deuterated chloroform (600 μL) was added for analysis by ^{1}H NMR spectroscopy and GC–MS.

Amorpha-4,11-diene (3)

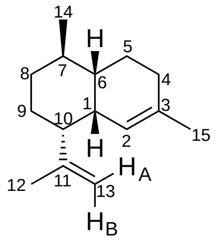

^{1}**H NMR** (500 MHz, $CDCl_3$): δ (ppm) 5.05 (1 H, bs, $C\underline{H}{=}CCH_3$), 4.87 (1 H, bs, $C{=}C\underline{H_A}$), 4.64 (1 H, bs, $C{=}C\underline{H_B}$), 2.55 (1 H, m, $CH_2CCHC\underline{H}$), 1.96-0.89 (11 H, m, $(C\underline{H_2})_2C\underline{H}C\underline{H}(C\underline{H_2})_2C\underline{H}$), 1.74 (3 H, s, $CH_2{=}CC\underline{H_3}$), 1.60 (3 H, s, $CH{=}CC\underline{H_3}$), 0.88 (3 H, d, J = 6.5, $CH_2CHC\underline{H_3}$). ^{13}**C NMR** (125 MHz, CDCl3): δ 148.2 ($CH_3\underline{C}{=}CH_2$), 134.8 ($CH_3\underline{C}{=}CH$), 121.1 ($CH_3C{=}\underline{C}H$), 110.0 ($CH_3C{=}\underline{C}H_2$), 47.9 ($CH2{=}C\underline{C}H$), 42.0 ($CH_3CCH_2CH_2\underline{C}H$), 37.8 ($C{=}CH\underline{C}H$), 35.6 ($CH_3CH\underline{C}H_2$), 26.7 ($CH_3C\underline{C}H_2$), 26.0 ($CH_3CHCH_2\underline{C}H_2$), 23.8 ($\underline{C}H_3C{=}CH$), 22.8 ($\underline{C}H_3C{=}CH_2$), 20.0 ($\underline{C}H_3CHCH_2$). **LRMS** ($EI^+$) *m/z*: 204.19 (63.5%, M^+), 189.16 (88.6), 175.15 (16.8), 162.14 (41.3), 147.12 (33.5), 133.10 (28.8), 119.08 (100), 105.07 (47.0), 93.07 (69.9), 79.05 (45.5), 67.05 (12.3).

8-Methoxy-γ-humulene (20)

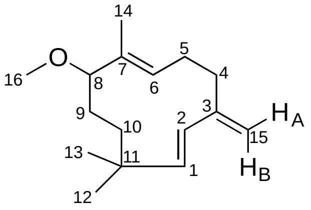

^{1}**H NMR** (500 MHz, $CDCl_3$): δ (ppm) 5.88 (1 H, d, J = 16.0, $CH{=}C\underline{H}$), 5.52 (1 H, t, J = 8.0, $CH_3C{=}C\underline{H}CH_2$), 5.48 (1 H, d, J = 16.0, $C\underline{H}{=}CH$), 4.89 (1 H, d, J = 2.0, $C\underline{H_A}{=}C$), 4.81 (1 H, d, J = 2.0, $C\underline{H_B}{=}C$), 3.30 (1 H, dd, J = 2.5 and 9.0, $OC\underline{H}$), 3.10 (3 H, s, $OC\underline{H_3}$), 1.71-1.25 (8 H, m, 2 x $C\underline{H_2}C\underline{H_2}$), 1.37 (3 H, s, $OCHCC\underline{H_3}$) 0.97 and 0.96 (2 x 3 H, 2 x s, 2 x $CH_2CC\underline{H_3}$). ^{13}**C NMR** (125 MHz, CDCl3): δ 148.8 ($\underline{C}{=}CH_2$), 143.0

($CH_3CCH{=}\underline{C}H$), 135.9 ($CH_3\underline{C}{=}CH$), 129.4 ($CH_3C{=}\underline{C}H$), 125.5 ($CH_3C\underline{C}H{=}CH$), 113.5 ($C{=}\underline{C}H_2$), 90.3 ($\underline{C}HO$), 55.7 ($O\underline{C}H_3$), 41.4 ($\underline{C}H_2$), 36.4 ($CH_2\underline{C}CH_3$), 31.3 ($\underline{C}H_2$), 30.3 ($C\underline{C}H_3$), 29.9 ($\underline{C}H_2$), 28.3 ($\underline{C}H_2$), 23.7 ($C\underline{C}H_3$), 11.6 ($CH{=}C\underline{C}H_3$). **LRMS** (EI^+) *m/z*: 234.20 (6.4%, M^+), 219.18 (4.2), 202.17 (86.3), 187.15 (100), 173.13 (33.2), 159.12 (83.1), 145.10 (51.5), 131.09 (88.5), 119.09 (45.8), 105.07 (76.5), 91.05 (96.7), 79.05 (52.6), 67.05 (14.5).

12-Methoxy-β-sesquiphellandrene (26)

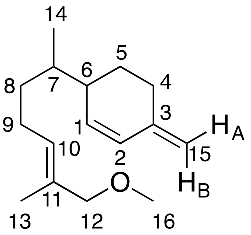

^{1}H NMR (400 MHz, $CDCl_3$): δ (ppm) 6.14 (1 H, d, *J* = 10.0, $CCH{=}C\underline{H}$), 5.66 (1 H, d, *J* = 11.5, $CC\underline{H}{=}CH$), 5.39 (1 H, t, *J* = 6.5, $C{=}C\underline{H}$), 4.75 (1 H, m, $C\underline{H_A}{=}C$), 4.74 (1 H, m, $C\underline{H_B}{=}C$), 3.79 (2 H, s, $OC\underline{H_2}$), 3.27 (3 H, s, $OC\underline{H_3}$), 2.46-1.19 (6 H, m, 2 x $C\underline{H_2}C\underline{H_2}$), 1.64 (3 H, s, $CH{=}CC\underline{H_3}$), 1.56 (1 H, m, $C\underline{H}CH_3$), 0.84 (3 H, d, *J* = 7.0, $CHC\underline{H_3}$). **LRMS** (EI^+) *m/z*: 234.20 (13.3%, M^+), 202.17 (41.6), 187.15 (37.2), 161.14 (29.9), 145.10 (47.6), 132.10 (53.8), 119.09 (100), 105.07 (55.1), 91.05 (74.7), 77.03 (40.5), 68.06 (53.3).

12-Methoxyzingiberene (27)

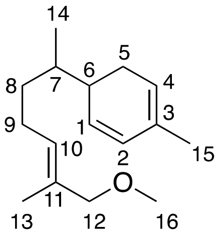

^{1}H NMR (400 MHz, $CDCl_3$): δ (ppm) 5.77 (1 H, d, *J* = 10.0, CCH=C<u>H</u>), 5.62 (1 H, m, CC<u>H</u>=CH), 5.45 (1 H, m, CHC=C<u>H</u>), 5.39 (1 H, t, *J* = 6.5, CH_2C=C<u>H</u>), 3.79 (2 H, s, OC<u>H</u>$_2$), 3.27 (3 H, s, OC<u>H</u>$_3$), 2.46-1.19 (6 H, m, C<u>H</u>$_2$C<u>H</u>$_2$ and CHC<u>H</u>$_2$), 1.71 (3 H, s, CHCC<u>H</u>$_3$), 1.64 (3 H, s, CH=CC<u>H</u>$_3$), 1.56 (1 H, m, C<u>H</u>CH_3), 0.87 (3 H, d, *J* = 7.0, CHC<u>H</u>$_3$). **LRMS** (EI$^+$) *m/z*: 234.20 (6.5%, M$^+$), 202.17 (20.5), 187.15 (17.4), 159.12 (14.0), 145.10 (29.9), 132.10 (57.0), 119.09 (100), 105.07 (36.7), 93.07 (91.5), 77.03 (35.7), 68.06 (24.3).

5. GC–MS

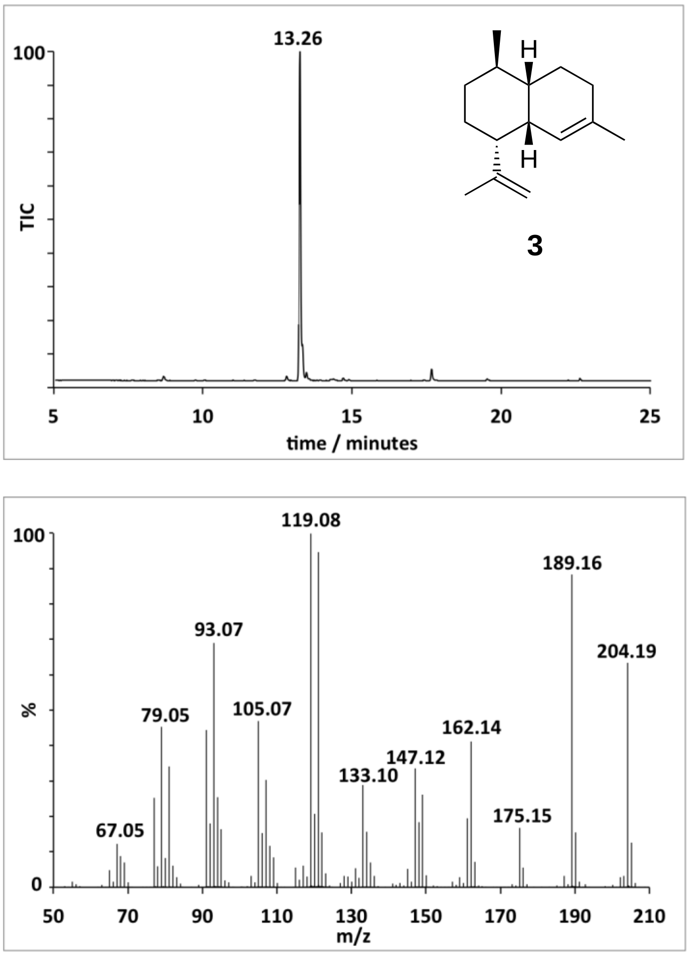

Figure S1. Gas chromatogram (top) and mass spectrum (bottom) of the organic soluble product isolated from an incubation of ADS with farnesyl diphosphate (FDP, **2**).

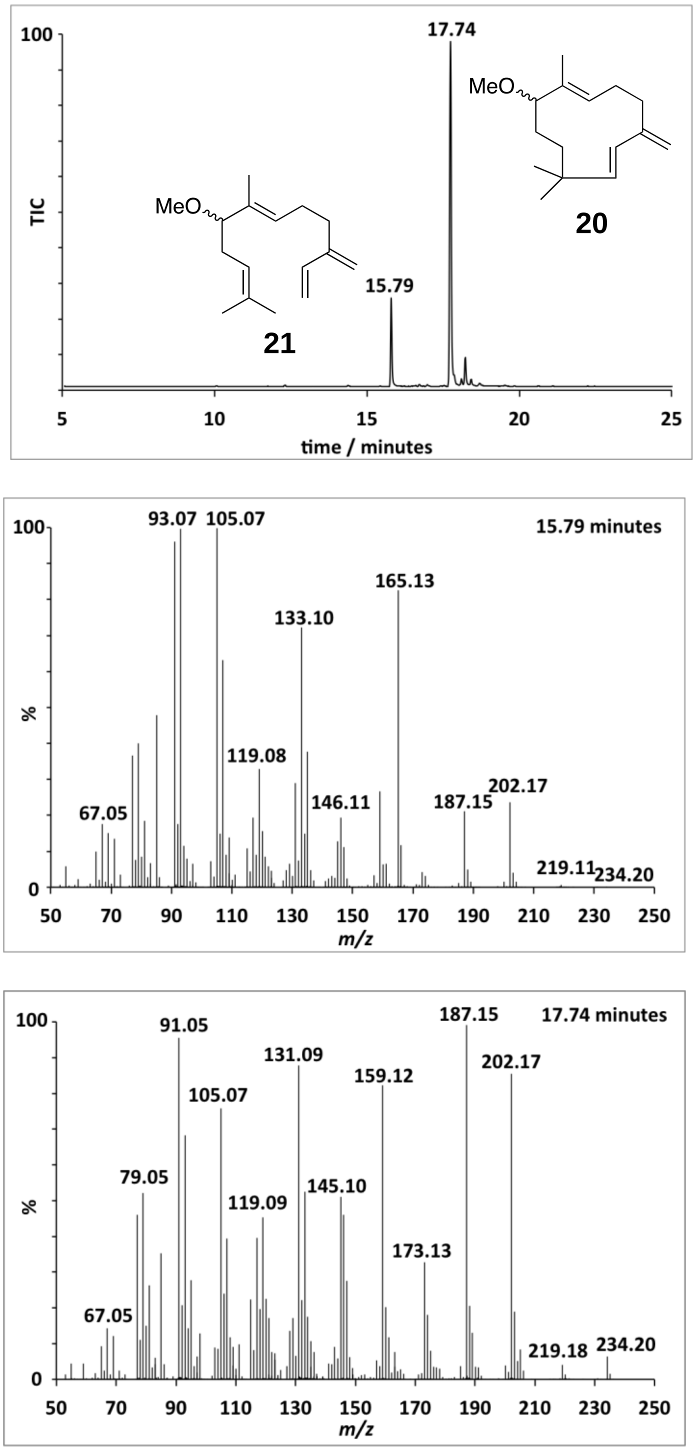

Figure S2. Gas chromatogram (top) and mass spectra (bottom) of the organic soluble products isolated from an incubation of ADS with 8-methoxyfarnesyl diphosphate (8-OMe FDP, **11**).

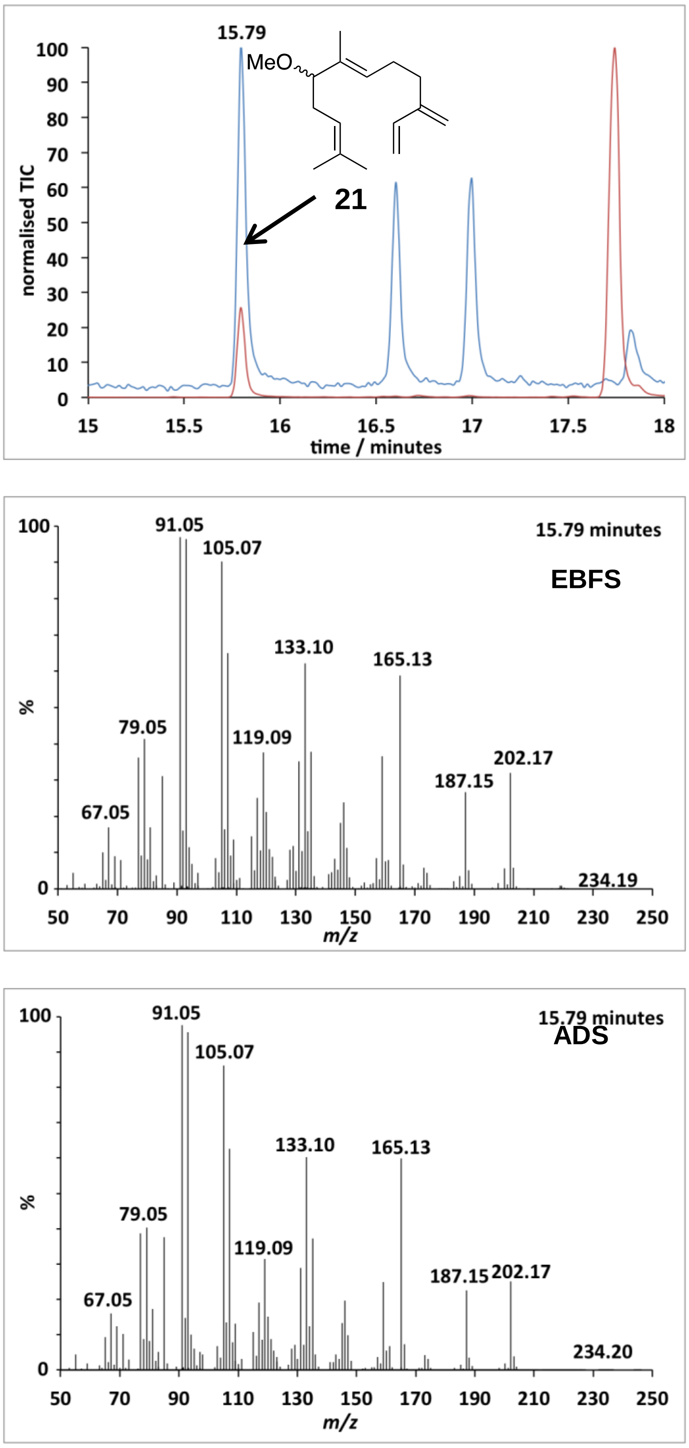

Figure S3. Overlaid gas chromatograms (top) of the organic soluble products isolated from an incubation of 8-methoxyfarnesyl diphosphate (8-OMe FDP, **11**) with EBFS (**blue**) and ADS (**red**) and superimposable mass spectra (bottom) of the respective co-eluting compounds (t_R = 15.79).

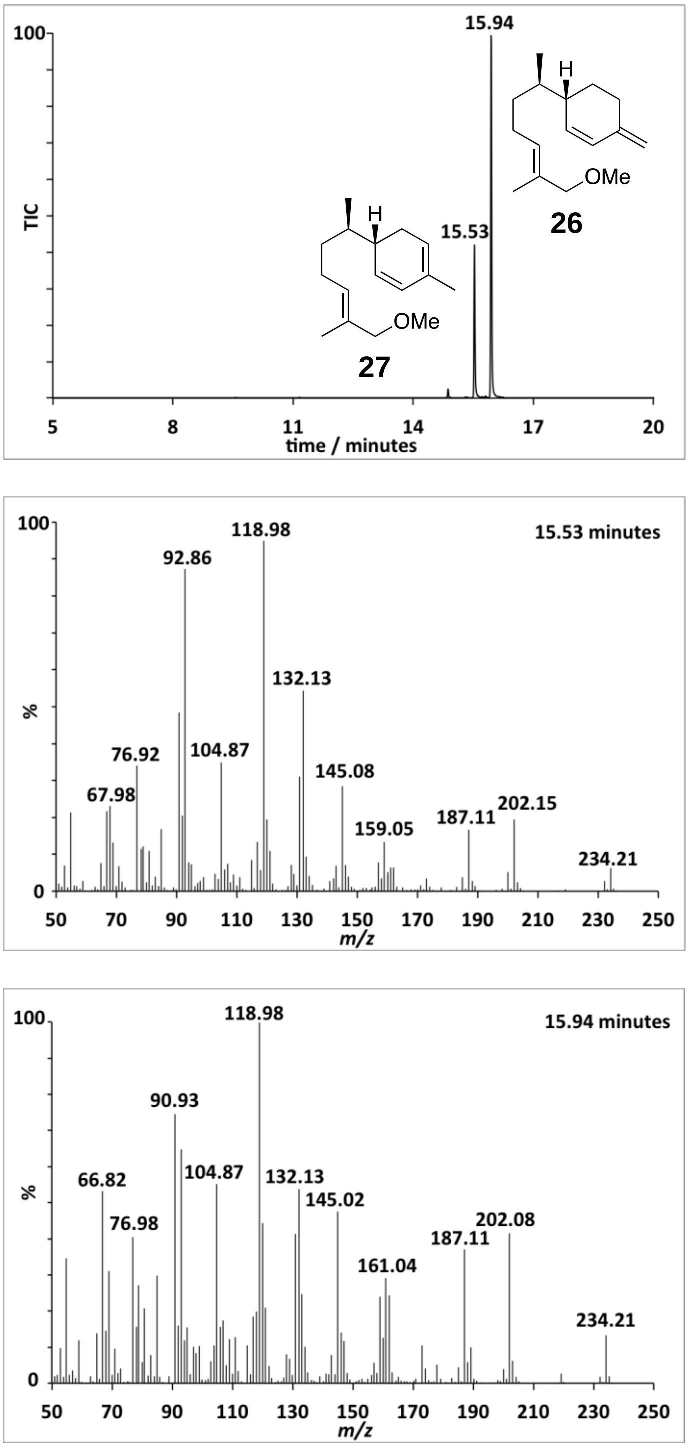

Figure S4. Gas chromatogram (top) and mass spectra (bottom) of the organic soluble products isolated from an incubation of ADS with 12-methoxyfarnesyl diphosphate (12-OMe FDP, **12**).

6. NMR SPECTRA

Figure S5. ^{1}H NMR spectrum ($CDCl_3$, 500 MHz) of farnesyl diphosphate (**2**).

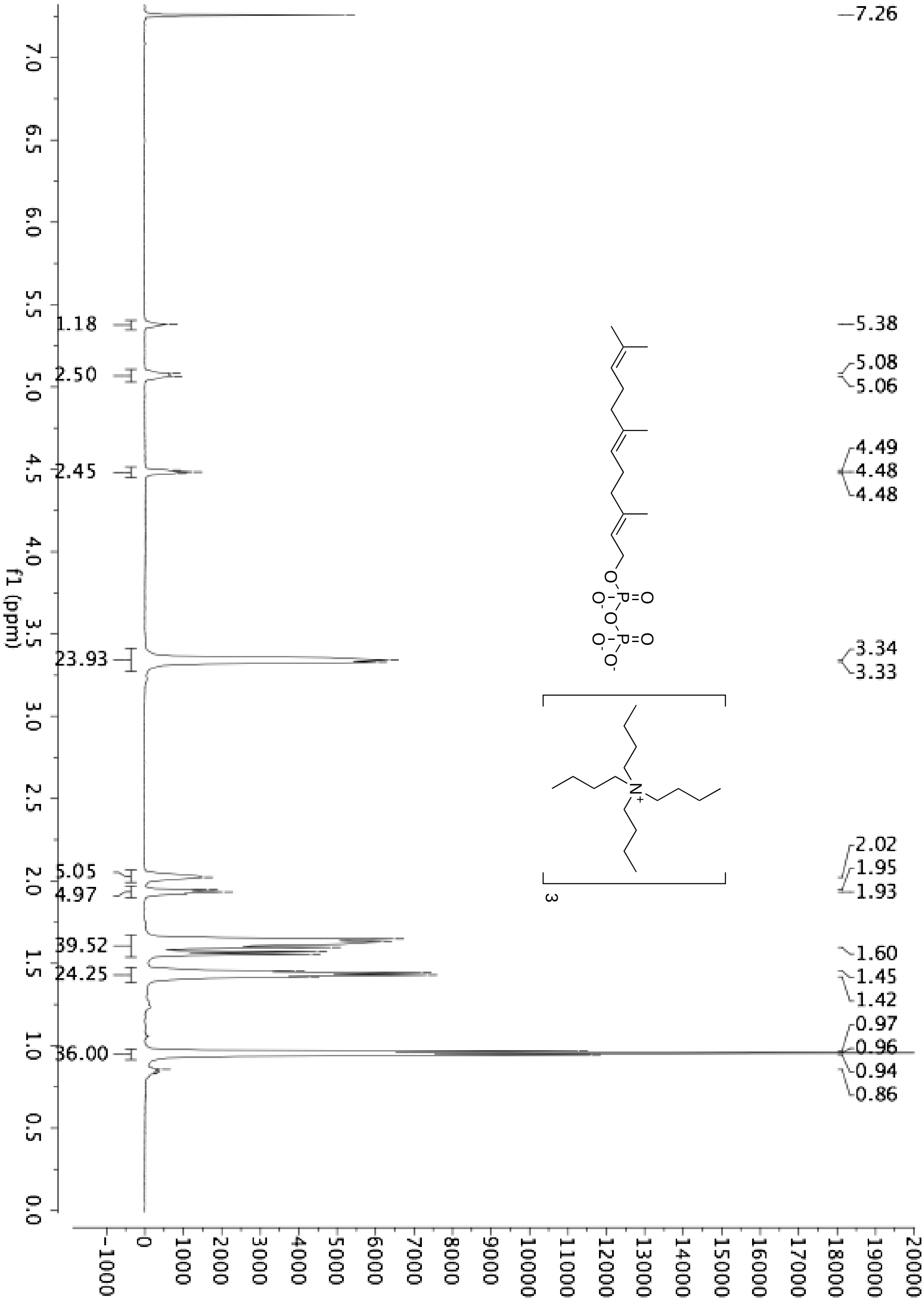

Figure S6. ^{1}H NMR spectrum ($CDCl_3$, 500 MHz) of 8-methoxyfarnesyl diphosphate (8-OMe FDP, **11**).

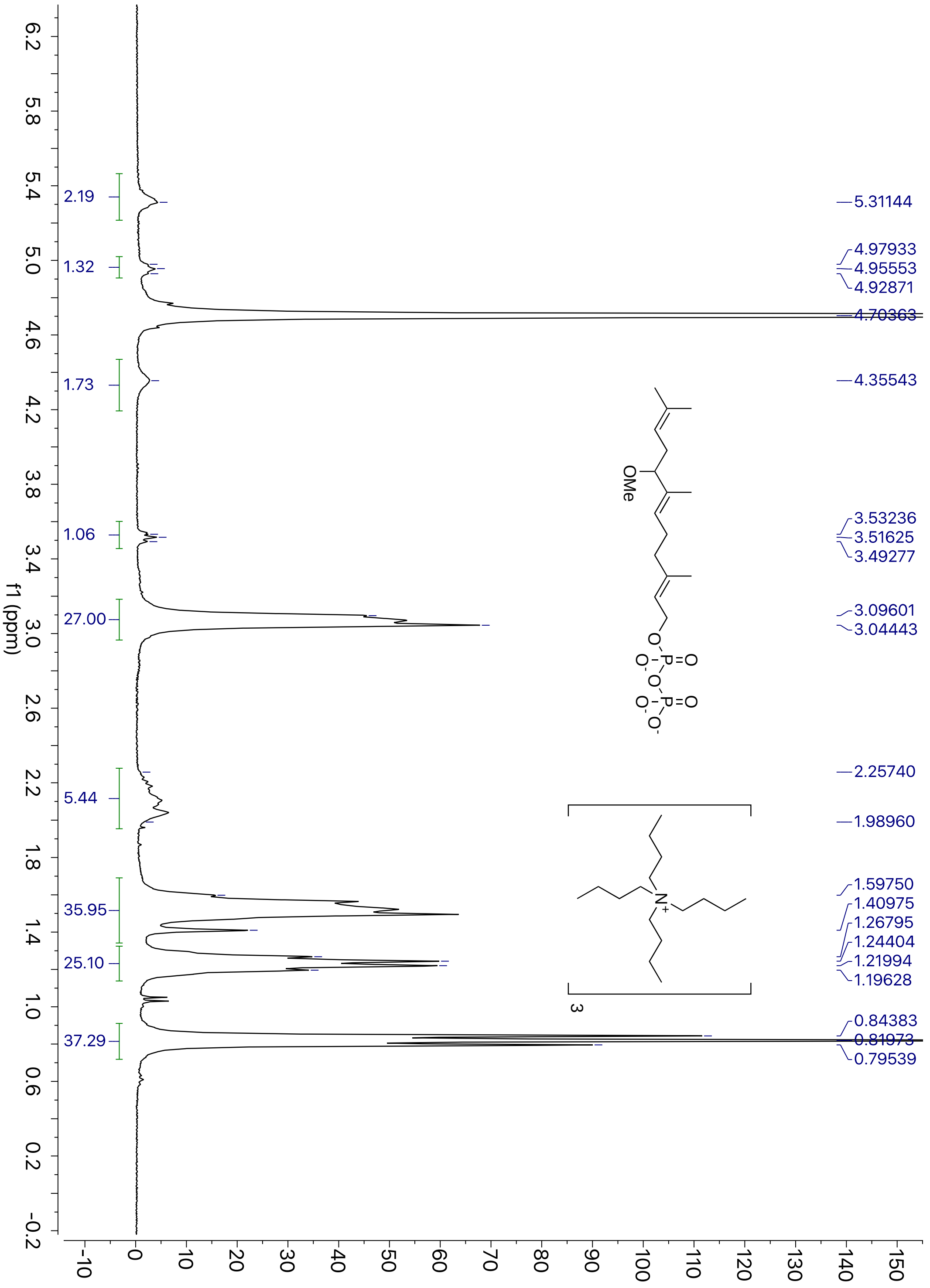

Figure S7. ^{31}P NMR spectrum ($CDCl_3$, 202 MHz) of 8-methoxyfarnesyl diphosphate (8-OMe FDP, **11**)

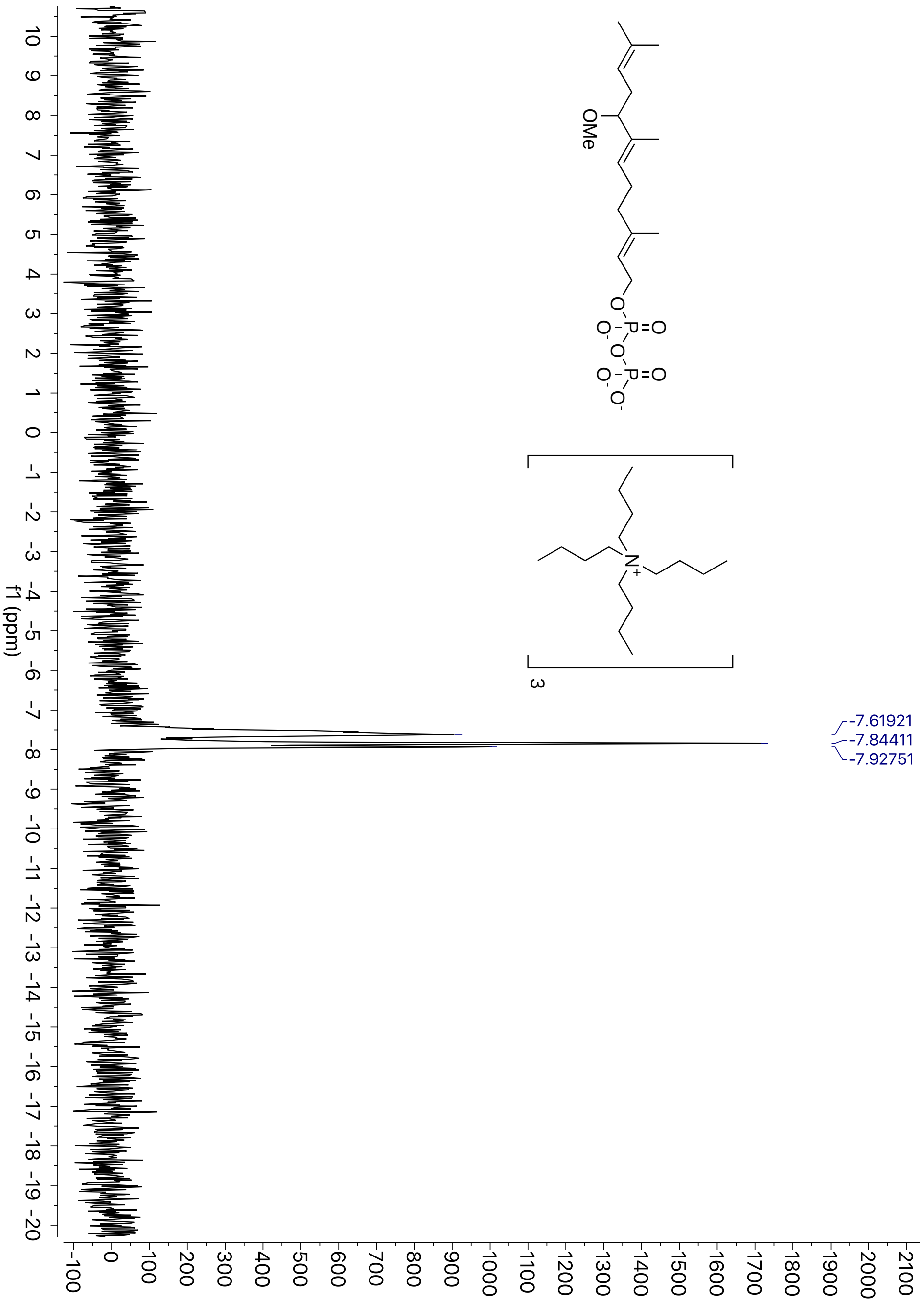

Figure S8. ^{1}H NMR spectrum ($CDCl_3$, 500 MHz) of 12-methoxyfarnesyl diphosphate (12-OMe FDP, **12**).

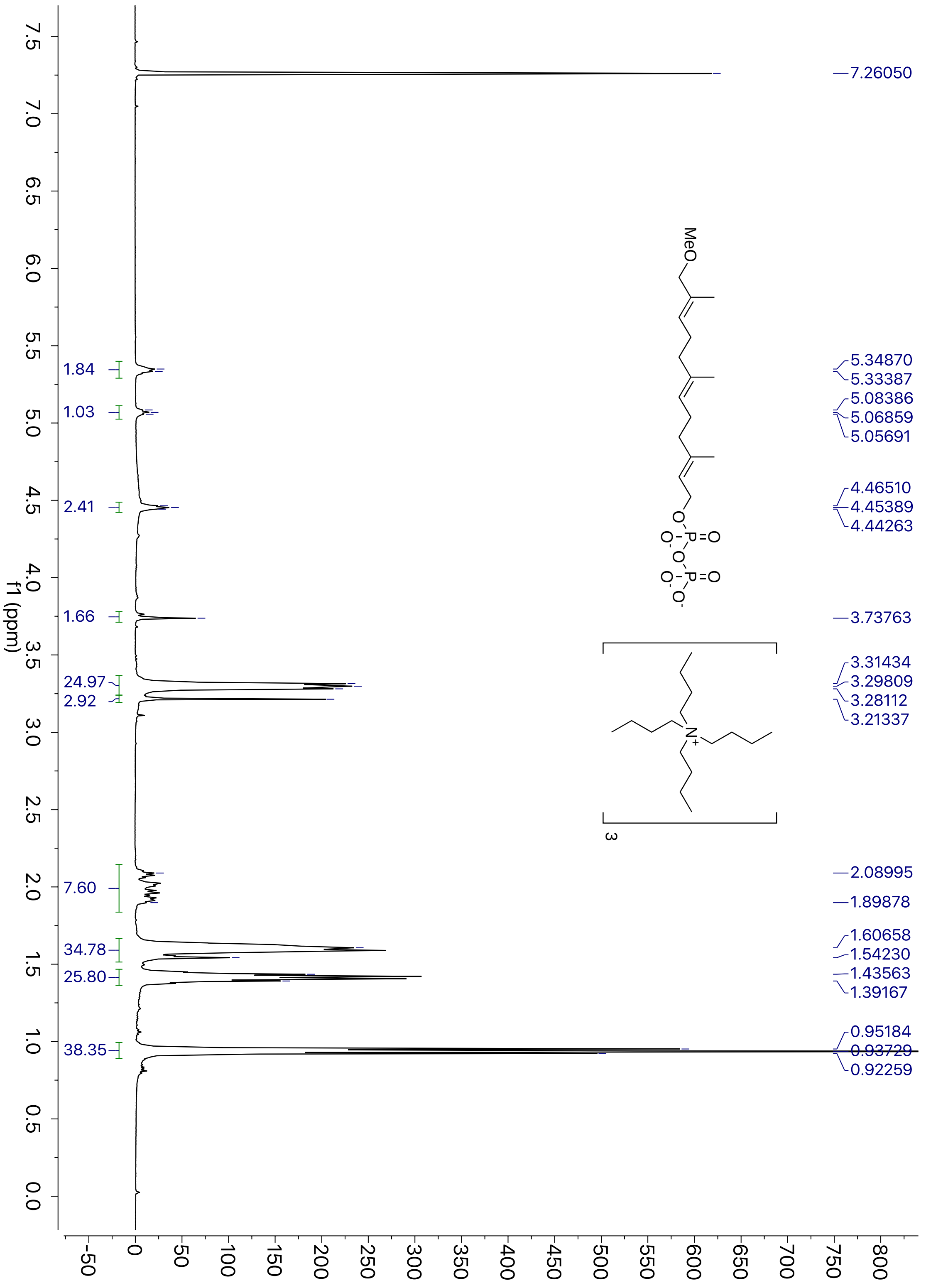

Figure S9. ^{31}P NMR spectrum ($CDCl_3$, 202 MHz) of 12-methoxyfarnesyl diphosphate (12-OMe FDP, **12**).

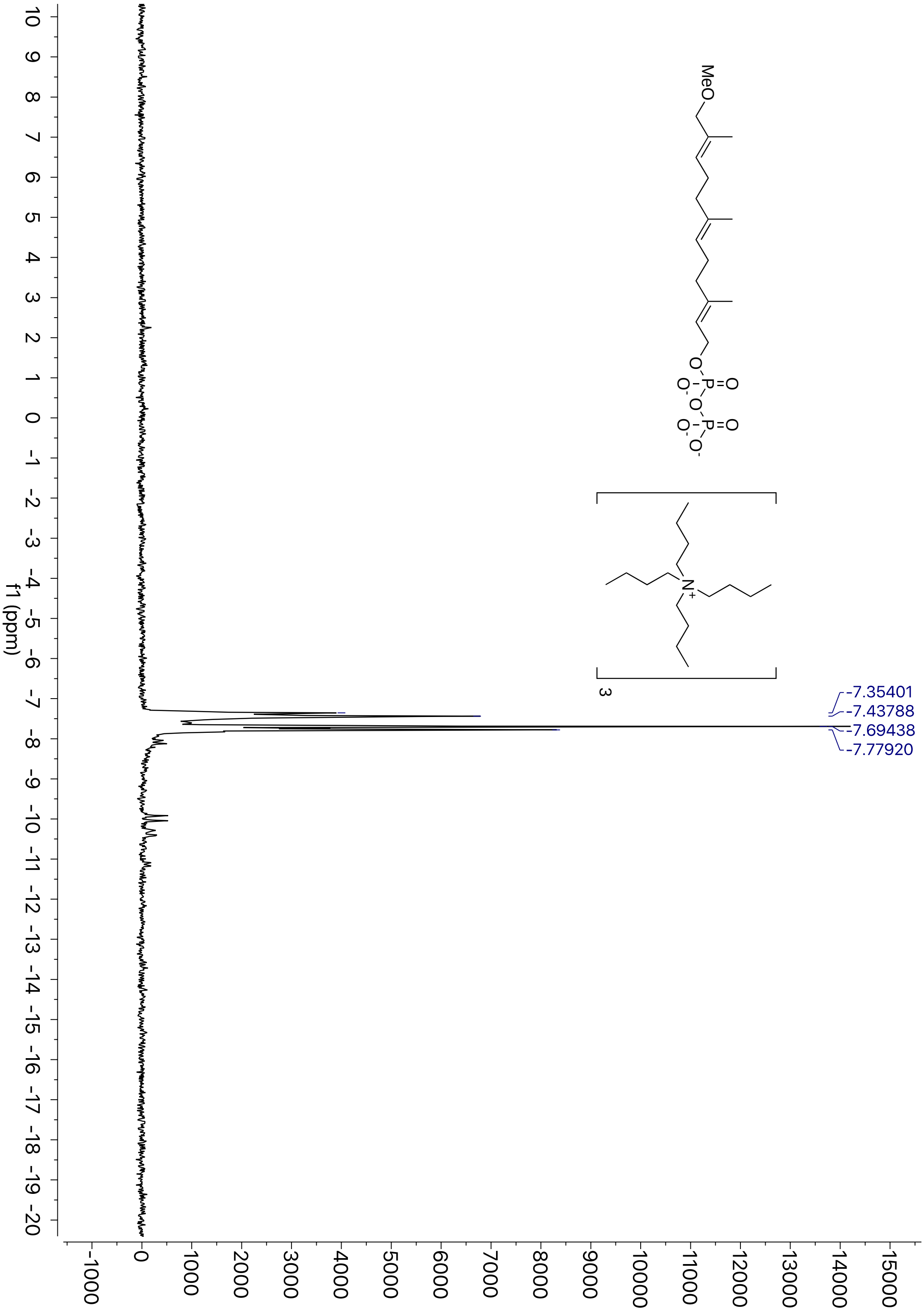

Figure S10. ^{1}H NMR spectrum ($CDCl_3$, 500 MHz) of the organic soluble product (amorpha-4,11-diene, **3**) isolated from an incubation of ADS with farnesyl diphosphate (FDP, **2**).

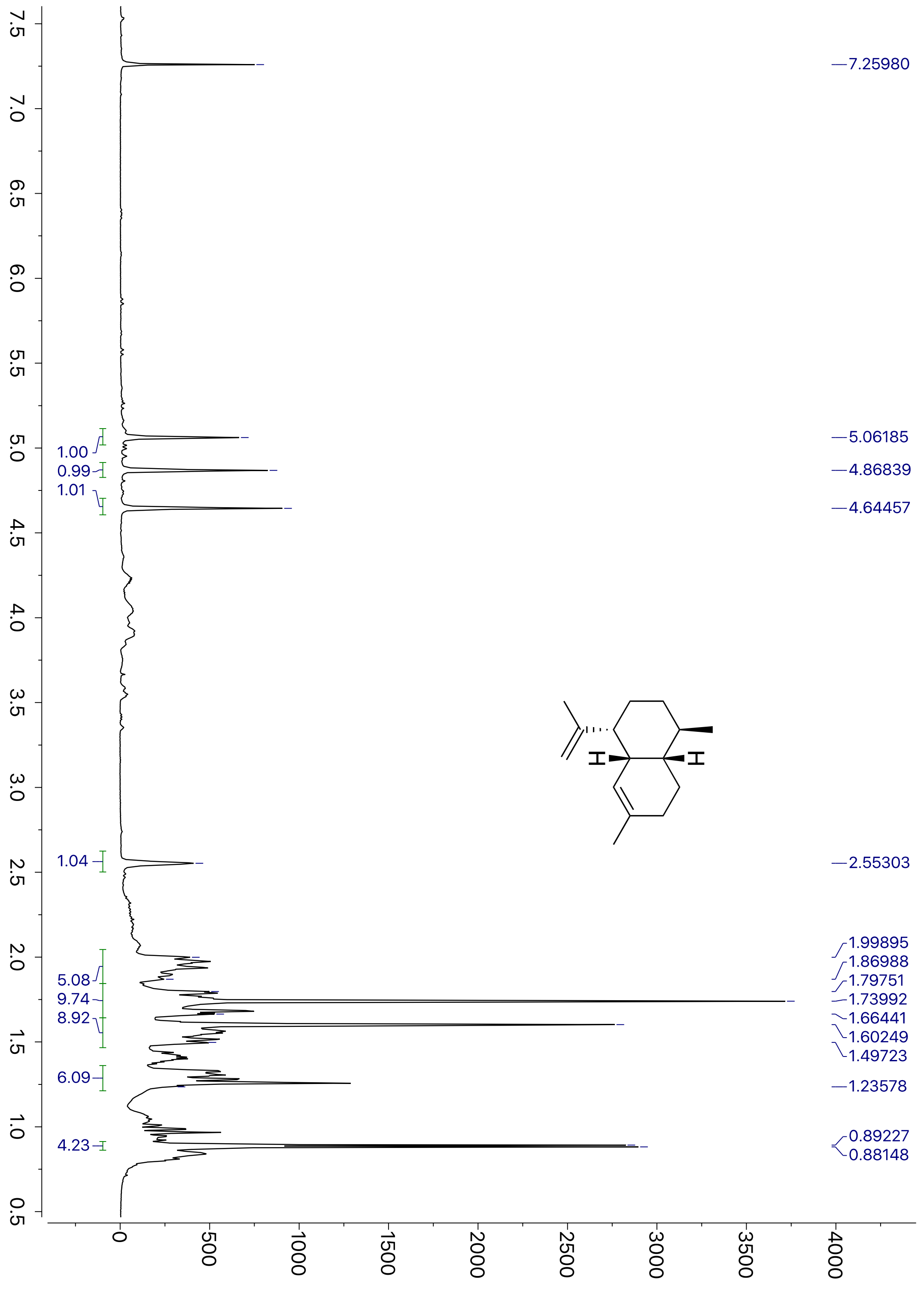

Figure S11. ^{13}C NMR spectrum ($CDCl_3$, 125 MHz) of the organic soluble product (amorphadiene, **3**) isolated from an incubation of ADS with farnesyl diphosphate (FDP, **2**).

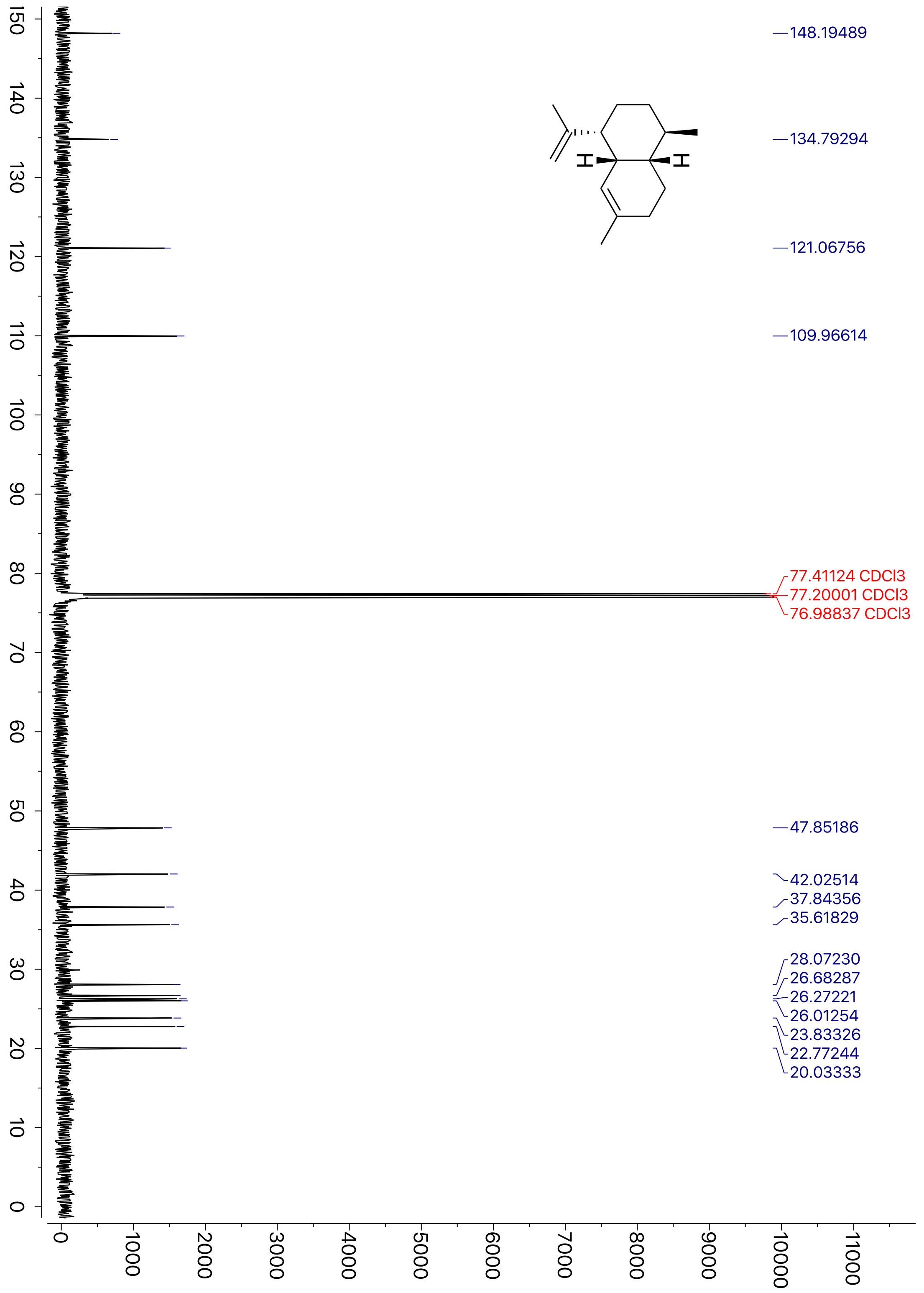

Figure S12. ^{1}H-^{13}C HSQC NMR spectrum ($CDCl_3$, 500 MHz) of the organic soluble product (amorphadiene, **3**) isolated from an incubation of ADS with farnesyl diphosphate (FDP, **2**).

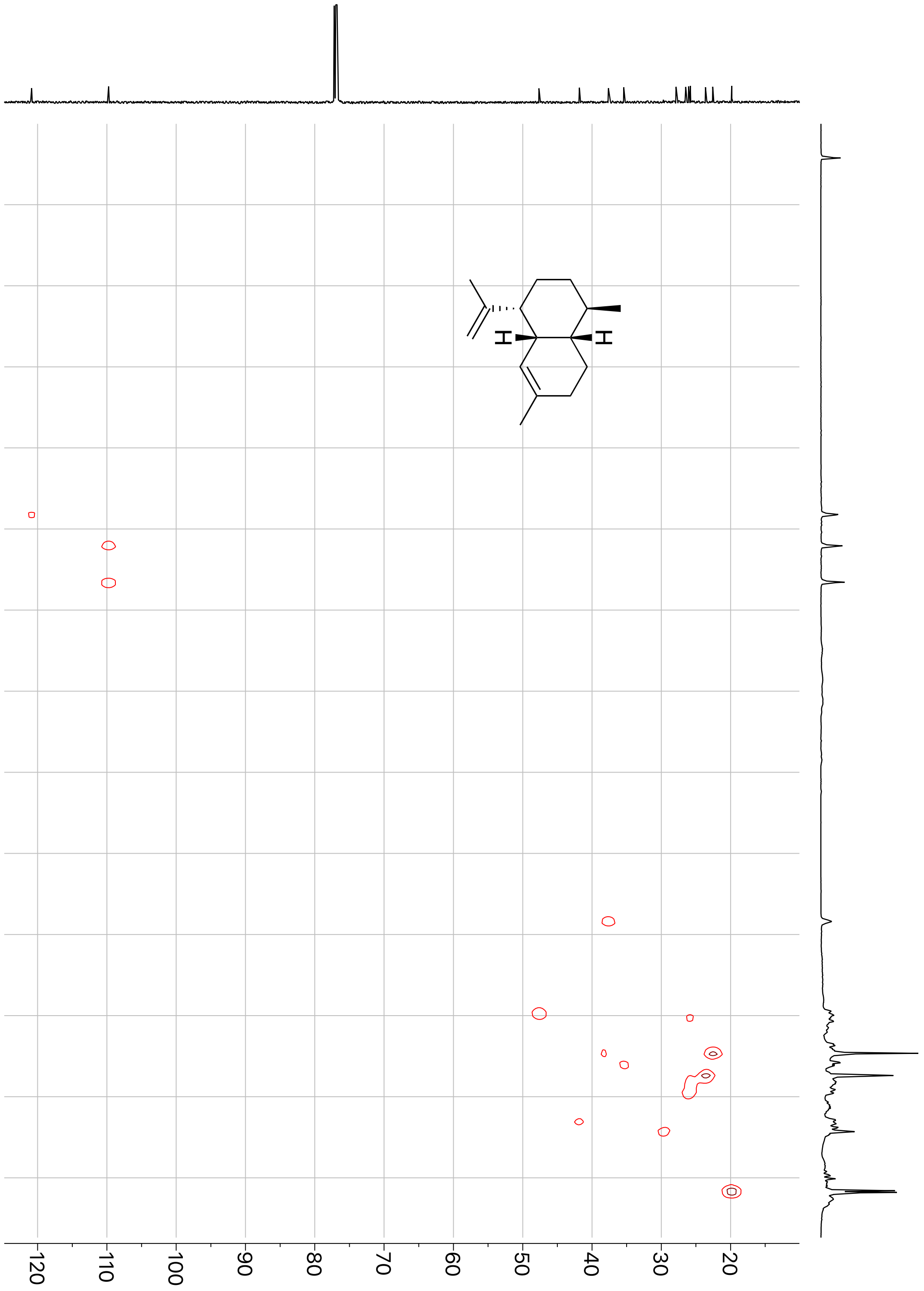

Figure S13. ^{1}H NMR spectrum ($CDCl_3$, 500 MHz) of the organic soluble products (8-methoxy-γ-humulene, **20** and (*E*)-8-methoxy-β-farnesene, **21**) isolated from an incubation of ADS with 8-methoxyfarnesyl diphosphate (8-OMe FDP, **11**).

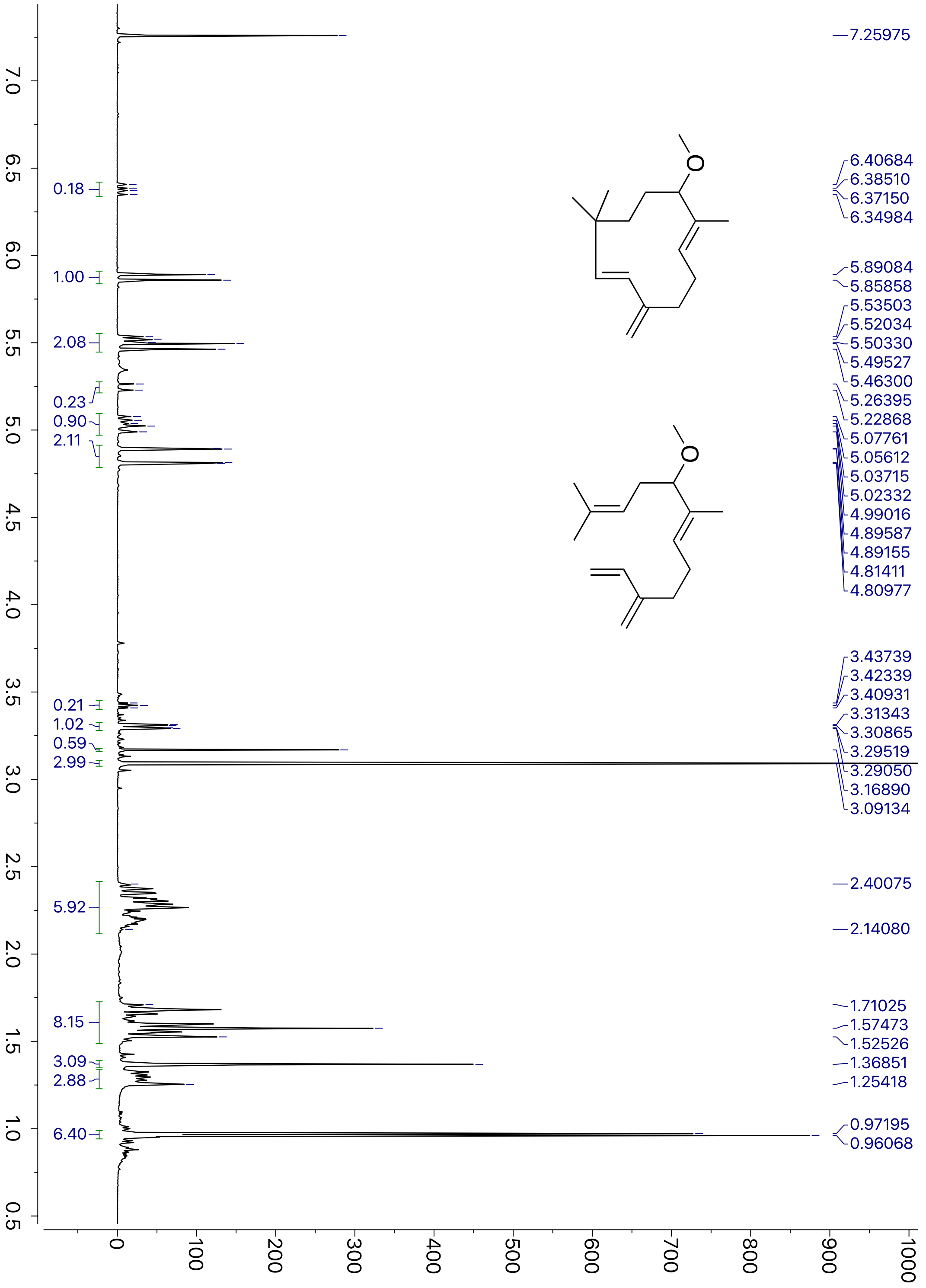

Figure S14. ^{13}C NMR spectrum ($CDCl_3$, 125 MHz) of the organic soluble products (8-methoxy-γ-humulene, **20** and (*E*)-8-methoxy-β-farnesene, **21**) isolated from an incubation of ADS with 8-methoxyfarnesyl diphosphate (8-OMe FDP, **11**).

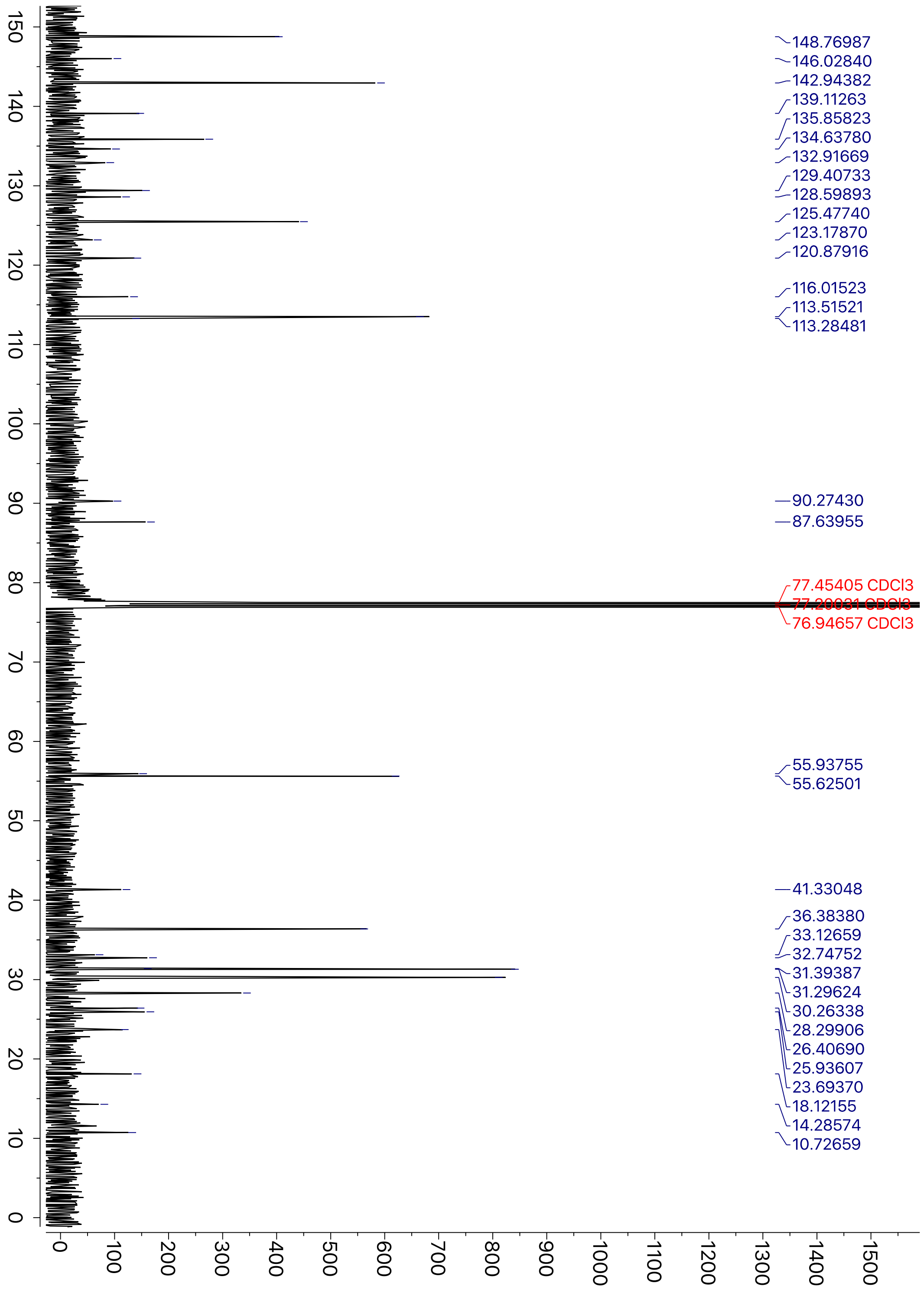

Figure S15. DEPT 135 ^{13}C NMR spectrum ($CDCl_3$, 125 MHz) of the organic soluble products (8-methoxy-γ-humulene, **20** and (*E*)-8-methoxy-β-farnesene, **21**) isolated from an incubation of ADS with 8-methoxyfarnesyl diphosphate (8-OMe FDP, **11**).

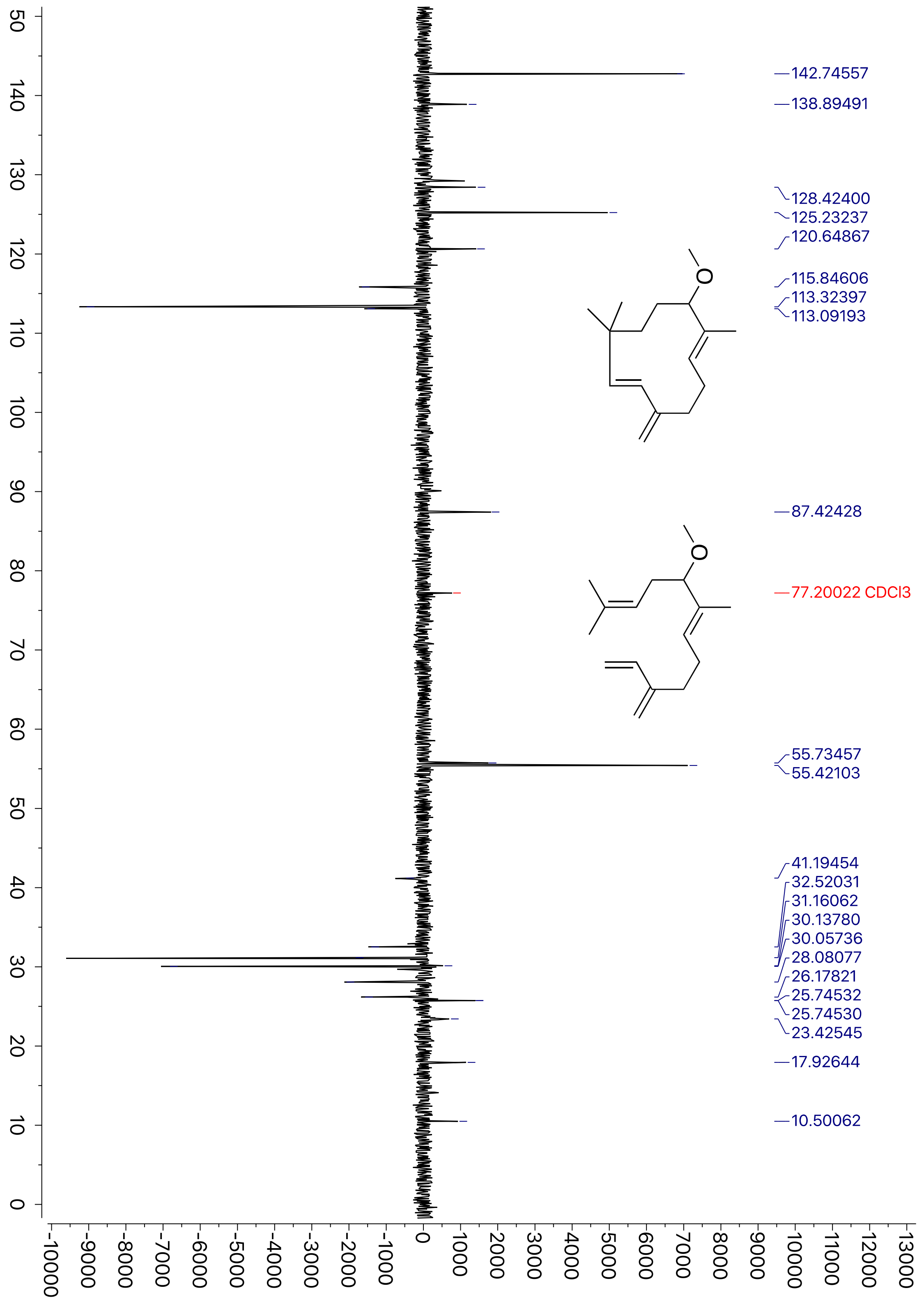

Figure S16. ^{1}H-^{13}C HSQC NMR spectrum ($CDCl_3$, 500 MHz) of the organic soluble products (8-methoxy-γ-humulene, **20** and (*E*)-8-methoxy-β-farnesene, **21**) isolated from an incubation of ADS with 8-methoxyfarnesyl diphosphate (8-OMe FDP, **11**).

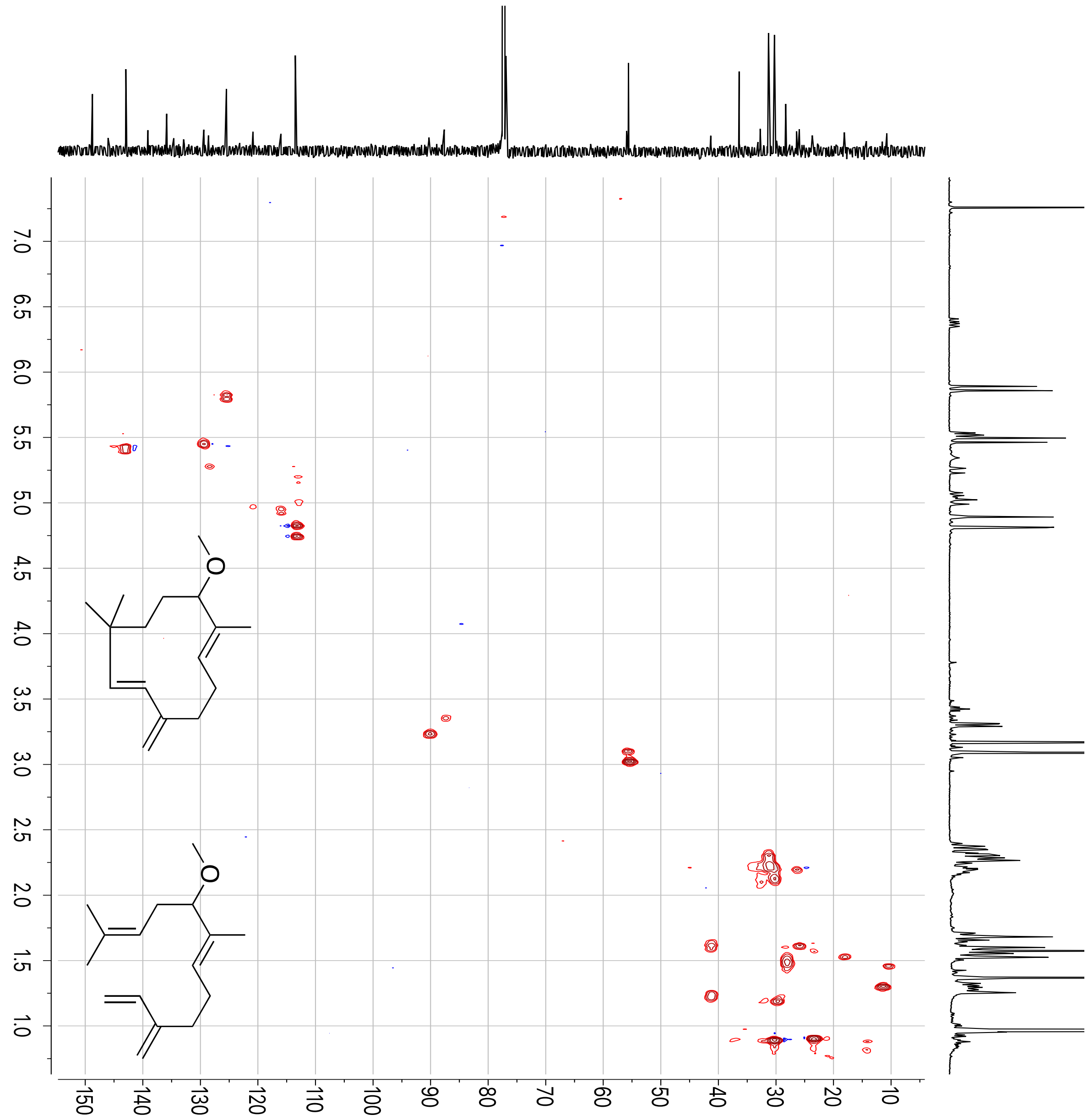

Figure S17. ^{1}H-^{13}C HMBC NMR spectrum ($CDCl_3$, 500 MHz) of the organic soluble products (8-methoxy-γ-humulene, **20** and (*E*)-8-methoxy-β-farnesene, **21**) isolated from an incubation of ADS with 8-methoxyfarnesyl diphosphate (8-OMe FDP, **11**).

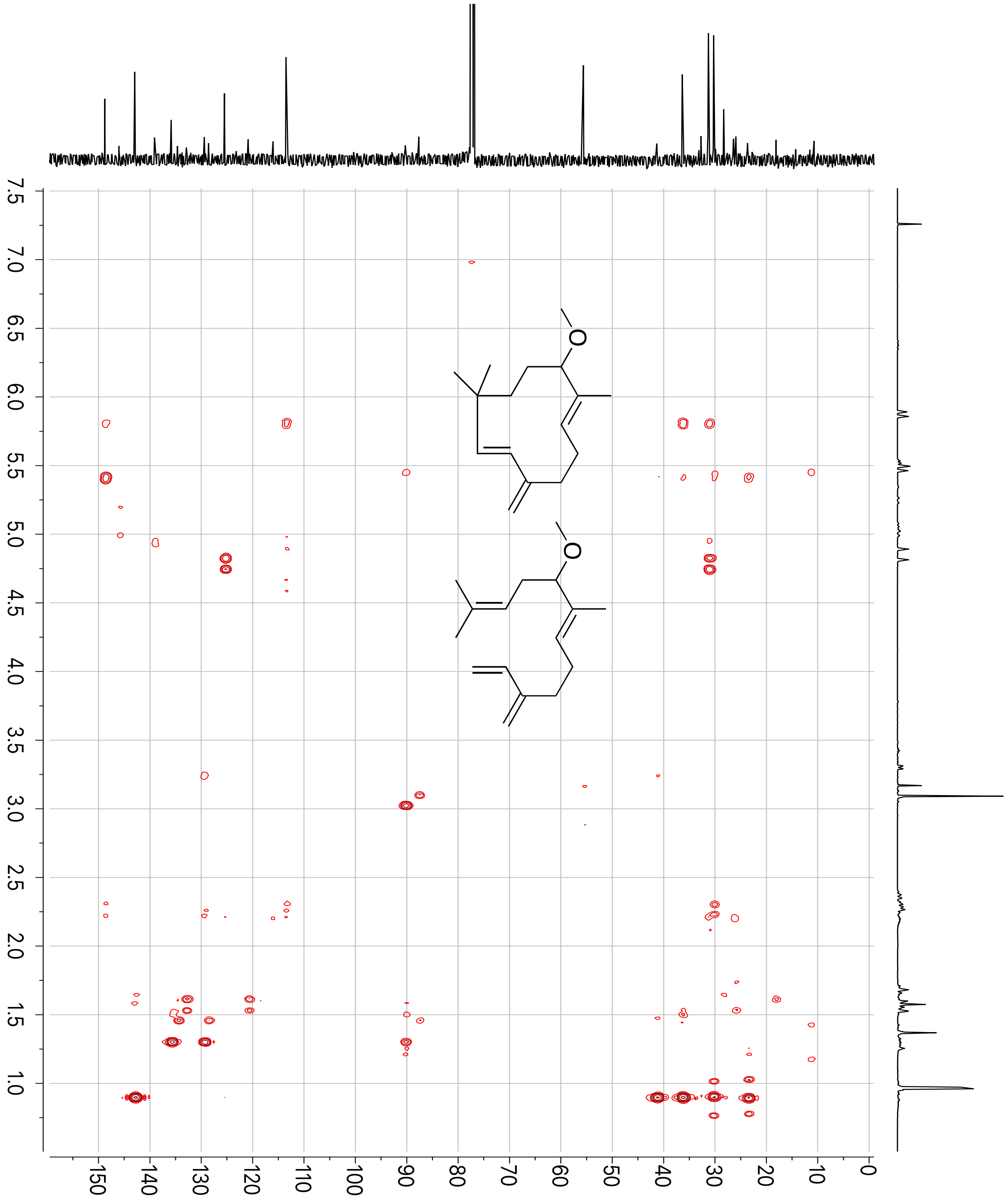

Figure S18. ^{1}H NMR spectrum ($CDCl_3$, 500 MHz) of the major organic soluble product (8-methoxy-γ-humulene, **20**) isolated from an incubation of ADS with 8-methoxyfarnesyl diphosphate (8-OMe FDP, **11**).

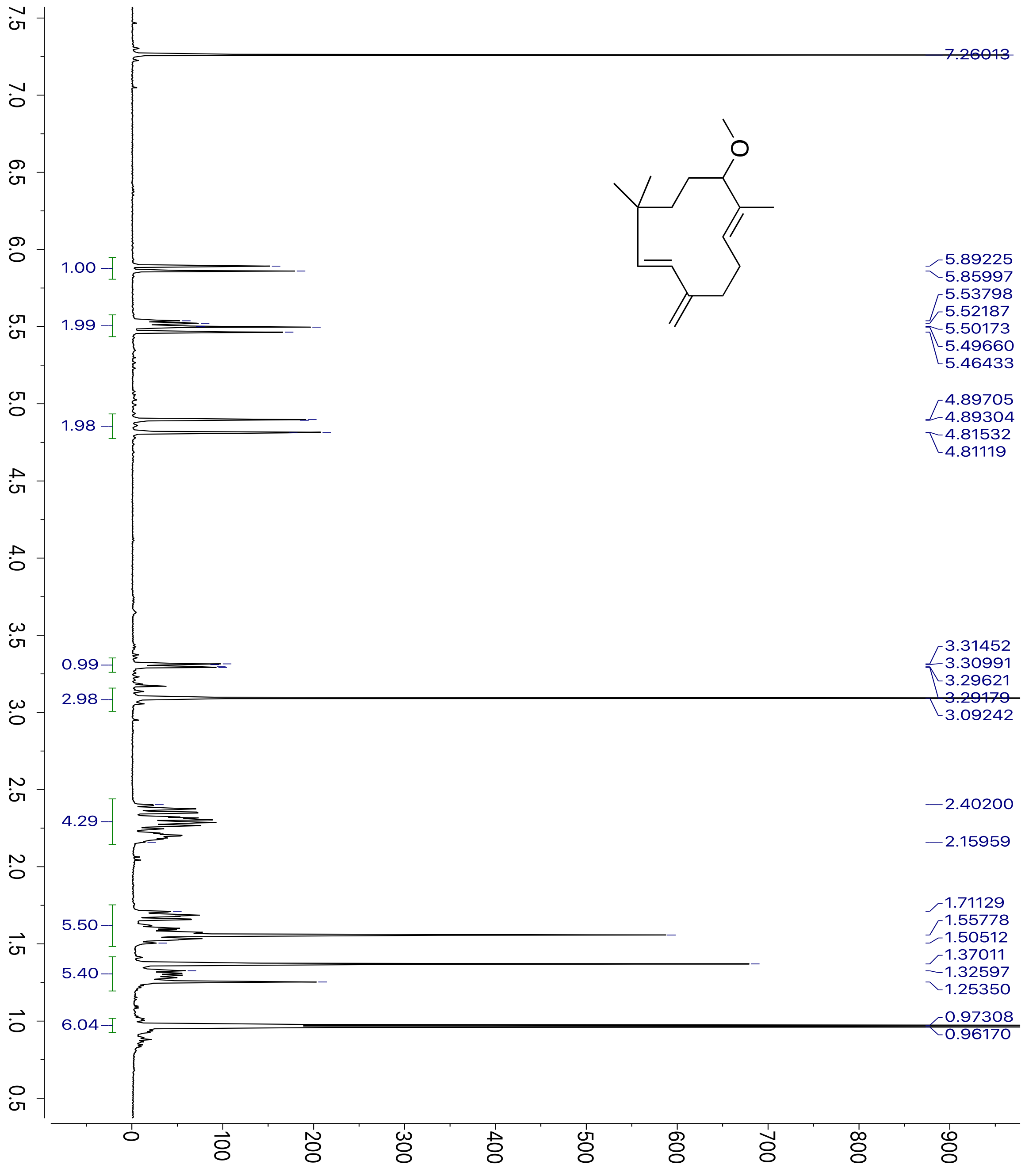

Figure S19. ^{13}C NMR spectrum ($CDCl_3$, 125 MHz) of the major organic soluble product (8-methoxy-γ-humulene, **20**) isolated from an incubation of ADS with 8-methoxyfarnesyl diphosphate (8-OMe FDP, **11**)

.

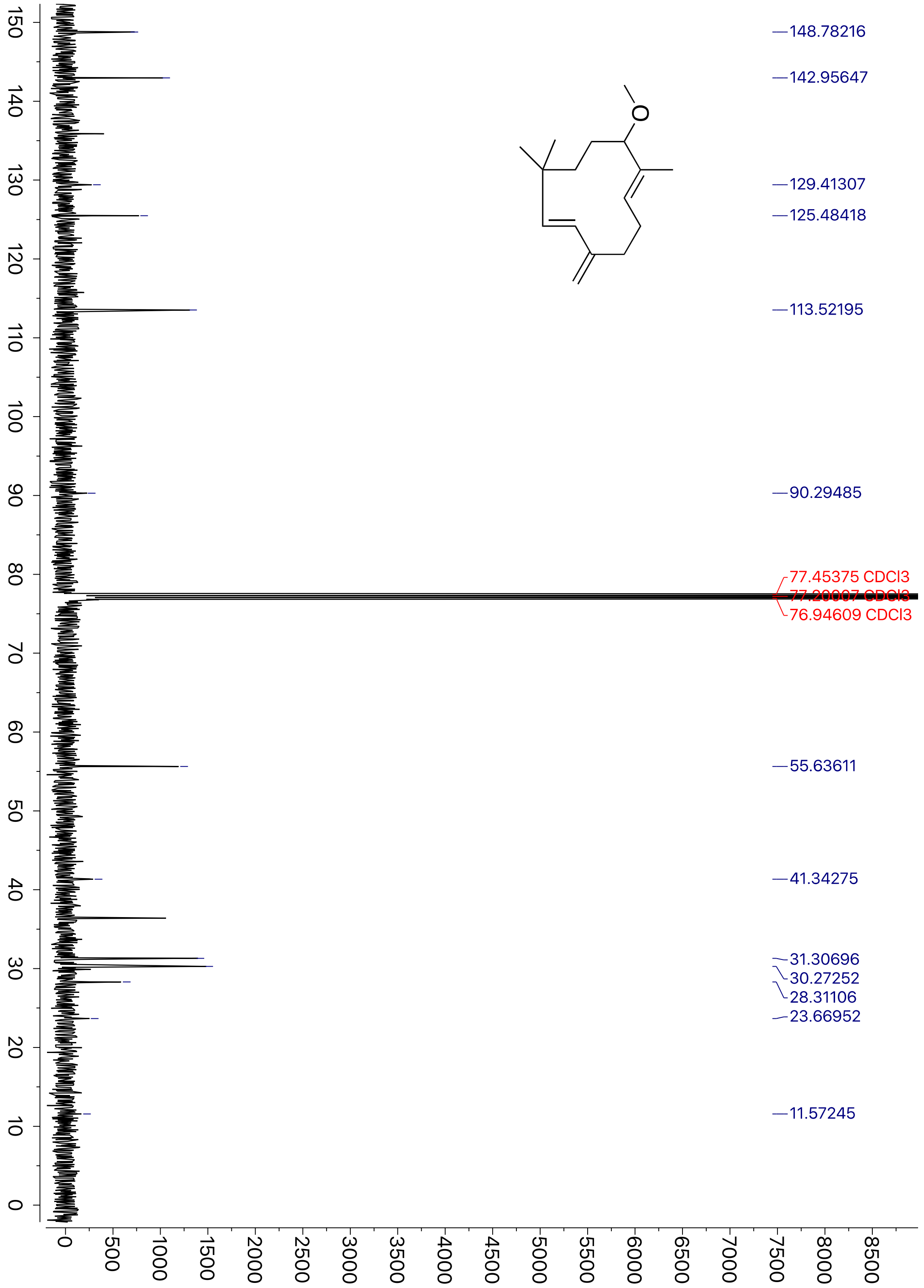

Figure S20. ^1H-^{13}C HSQC NMR spectrum ($CDCl_3$, 500 MHz) of the major organic soluble product (8-methoxy-γ-humulene, **20**) isolated from an incubation of ADS with 8-methoxyfarnesyl diphosphate (8-OMe FDP, **11**).

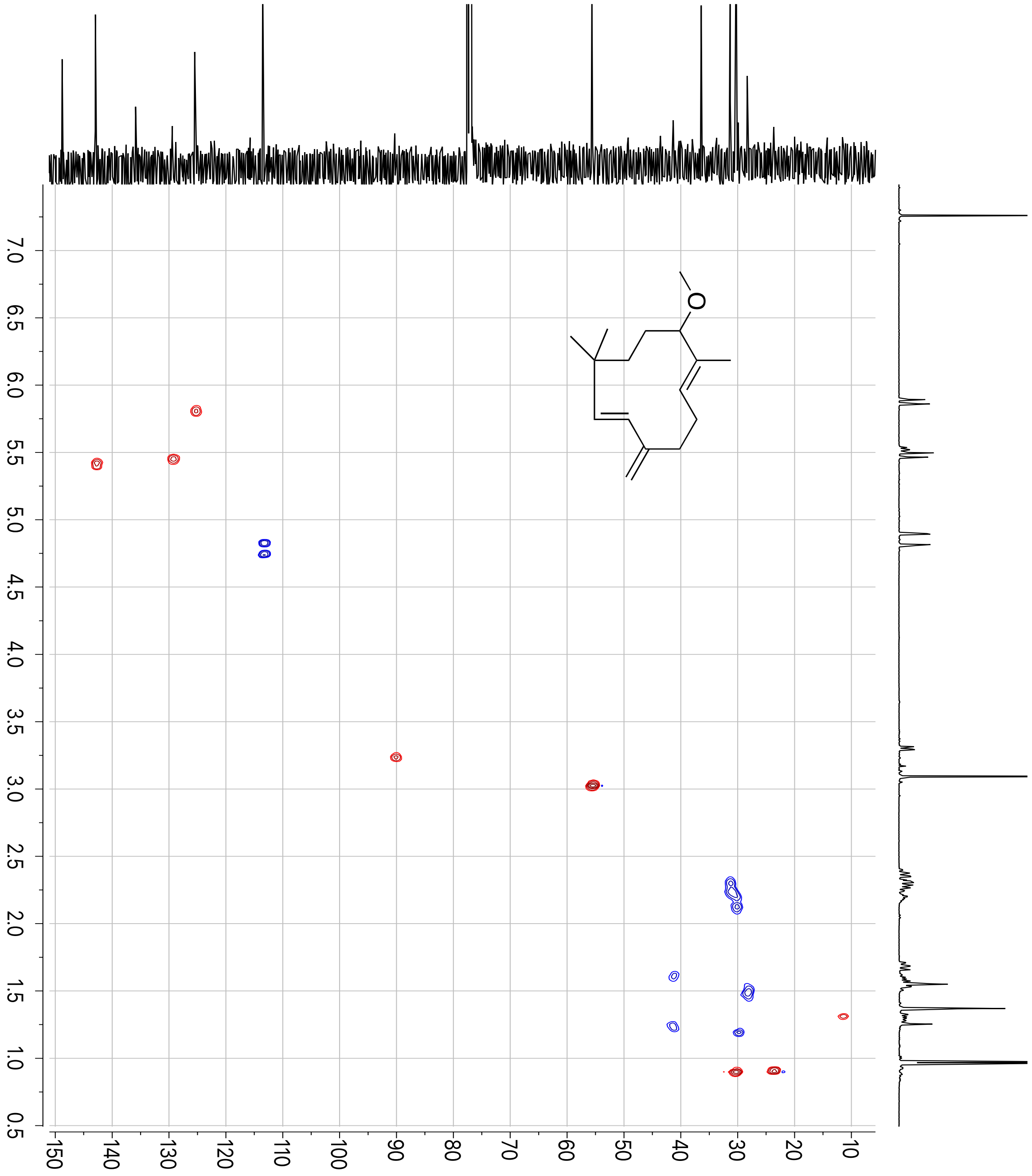

Figure S21. ^{1}H-^{13}C HMBC NMR spectrum ($CDCl_3$, 500 MHz) of the major organic soluble product (8-methoxy-γ-humulene, **20**) isolated from an incubation of ADS with 8-methoxyfarnesyl diphosphate (8-OMe FDP, **11**).

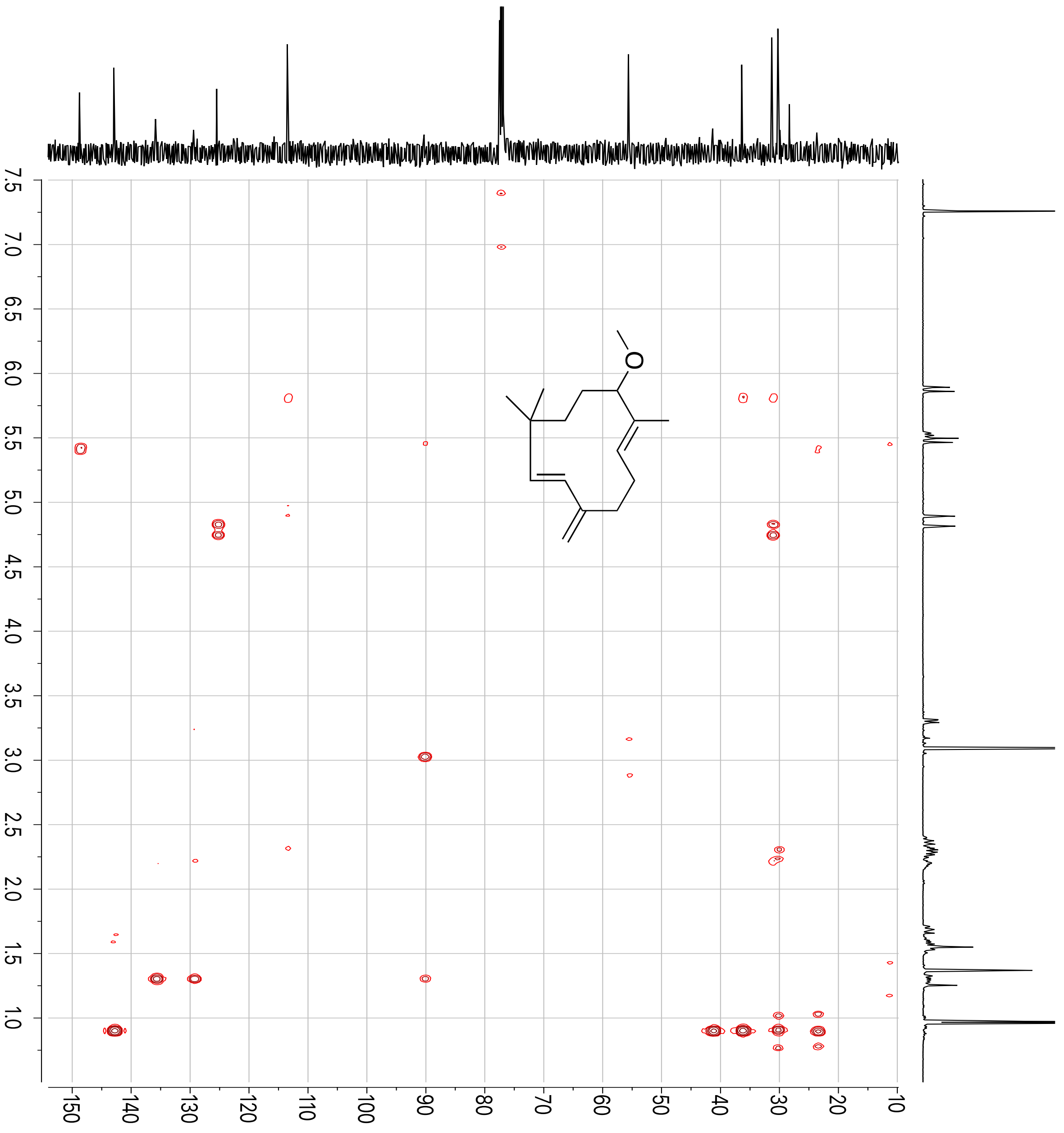

Figure S22. ^{1}H NMR spectrum ($CDCl_3$, 500 MHz) of the organic soluble products (12-methoxy-β-sesquiphellandrene, **26** and 12-methox-zingiberene, **27**) isolated from an incubation of ADS with 8-methoxyfarnesyl diphosphate (12-OMe FDP, **12**).

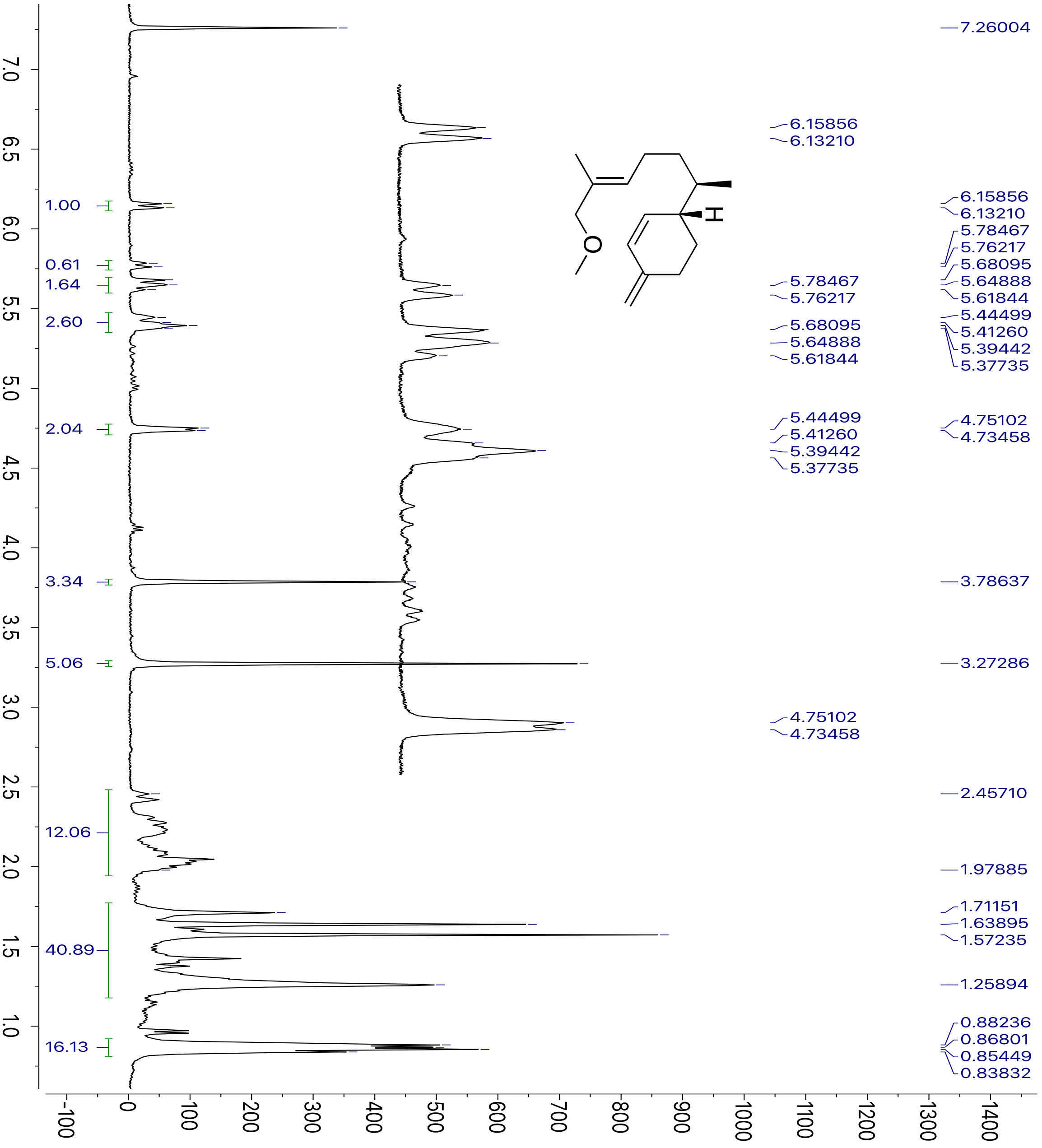

Figure S23. DEPT 135 ^{13}C NMR spectrum ($CDCl_3$, 125 MHz) of the organic soluble products (12-methoxy-β-sesquiphellandrene, **26** and 12-methoxyzingiberene, **27**) isolated from an incubation of ADS with 8-methoxyfarnesyl diphosphate (12-OMe FDP, **12**).

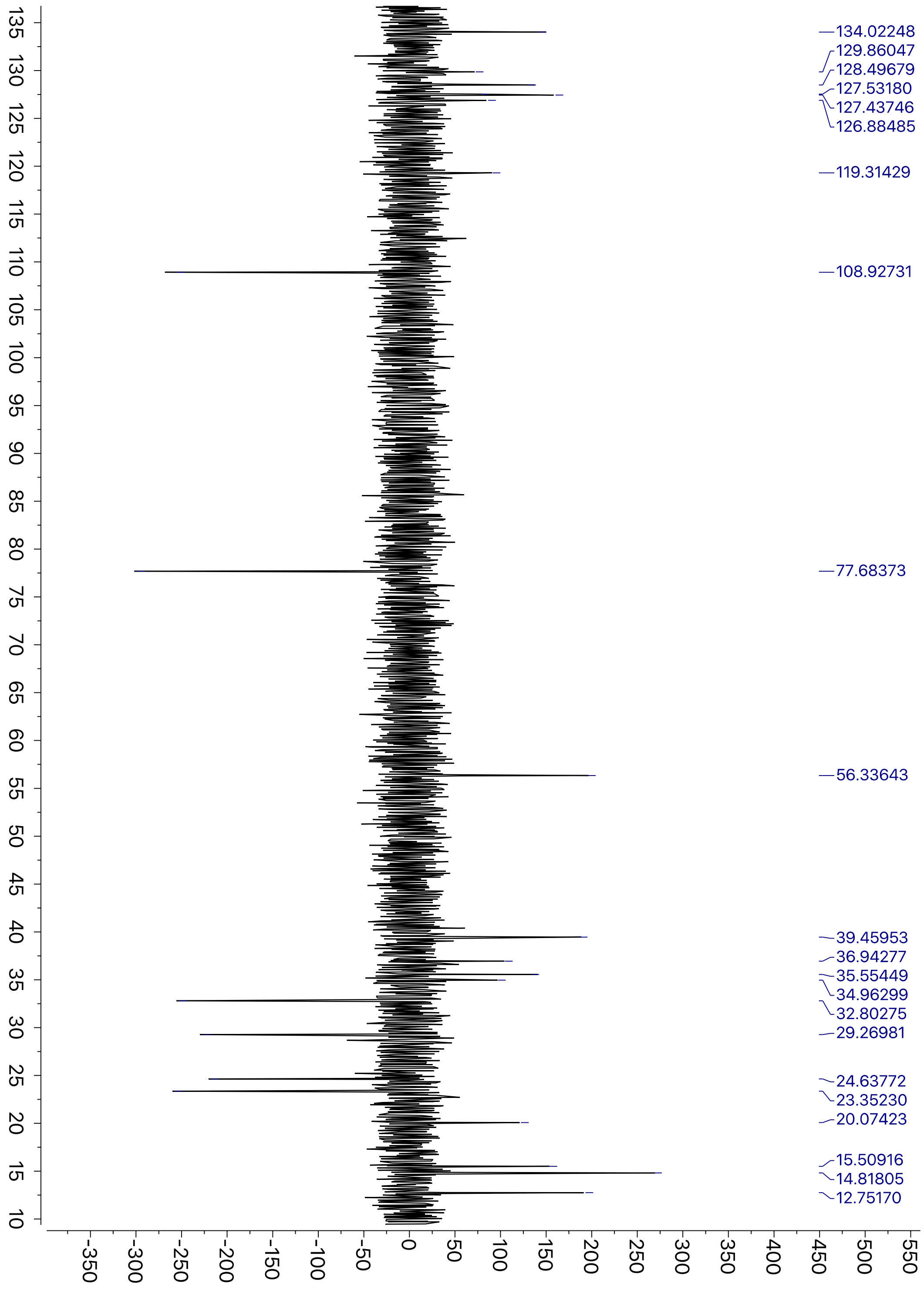

Figure S24. ^{1}H-^{13}C HSQC NMR spectrum (CDCl$_3$, 500 MHz) of the major organic soluble product (12-methoxy-β-sesquiphellandrene, **26** and 12-methoxyzingiberene, **27)** isolated from an incubation of ADS with 8-methoxyfarnesyl diphosphate (12-OMe FDP, **12**).

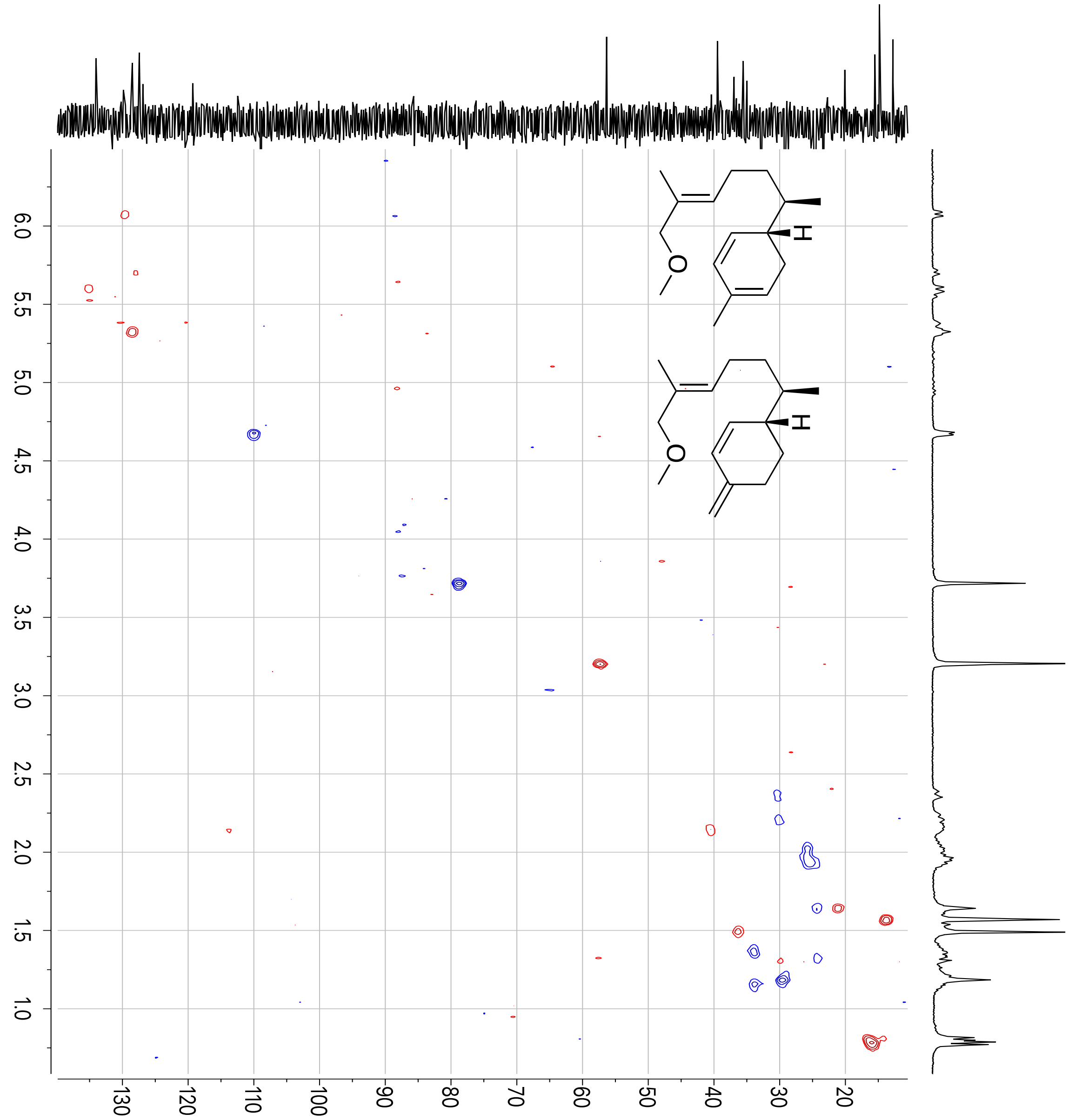

Figure S25. ^1H-^{13}C HMBC NMR spectrum ($CDCl_3$, 500 MHz) of the major organic soluble product (12-methoxy-β-sesquiphellandrene, **26** and 12-methoxyzingiberene, **27**) isolated from an incubation of ADS with 8-methoxyfarnesyl diphosphate (12-OMe FDP, **12**).

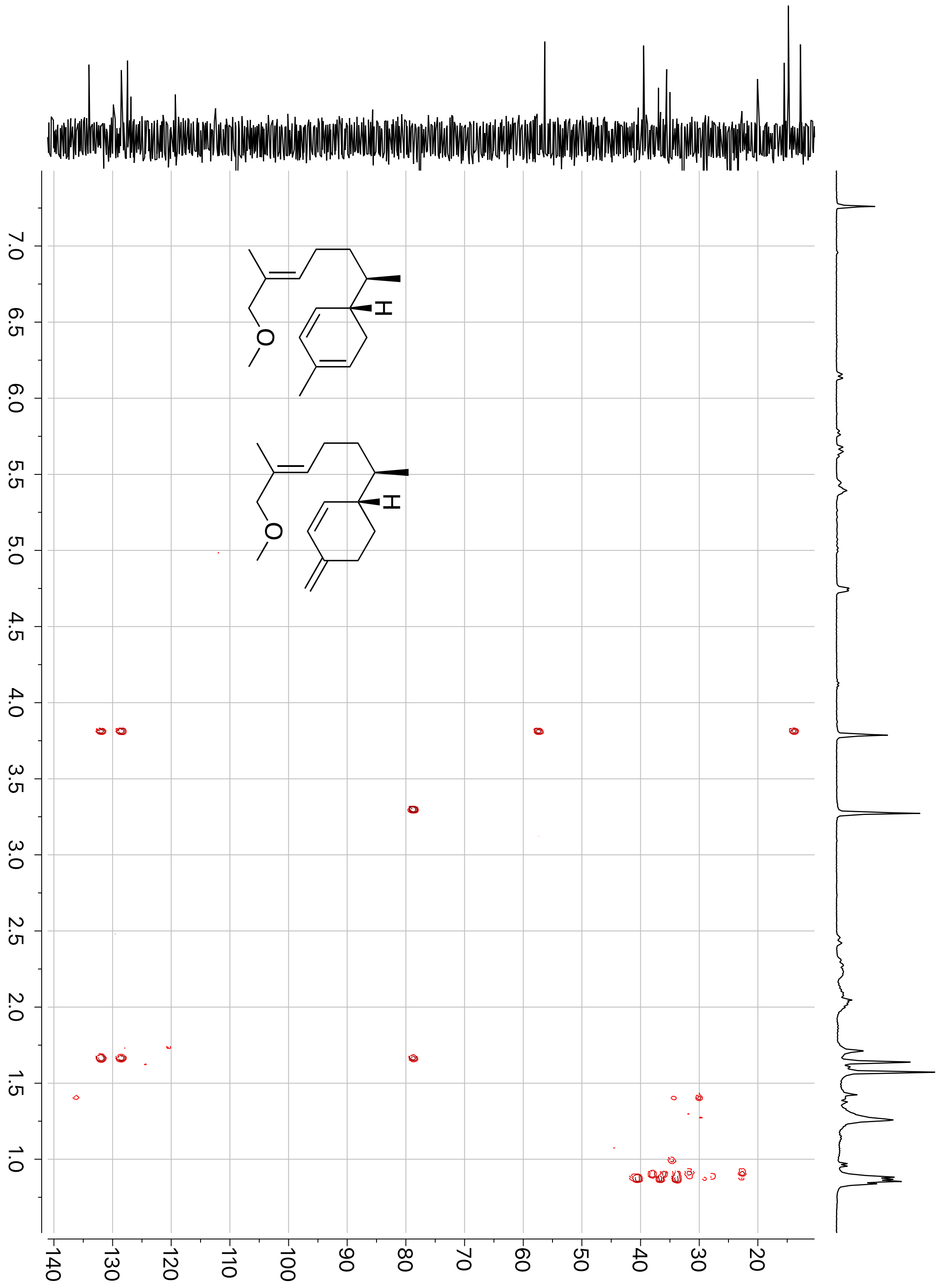

7. References

[1] Still, W. C.; Kahn, M.; Mitra, A. *J. Org. Chem.* **1978**, *43*, 2923–2925.

[2] Davisson, V. J.; Woodside, A. B.; Neal, T. R.; Stremler, K. E.; Muehlbacher, M.; Poulter, C. D. *J. Org. Chem.* **1986**, *51*, 4768–4779.

[3] Demiray, M.; Tang, X.; Faraldos, J. A.; Wirth, T.; Allemann, R. K.; *Angew. Chem. Int. Ed.* **2017**, *56*, 4347-4350.

[4] Bradford, M. M. *Anal. Biochem.* **1976**, *72*, 248–54.

[5] Zor, T.; Selinger, Z. *Anal. Biochem.* **1996**, *236*, 302–308.
